# The Effectiveness of Geographic Information Systems in Social Studies Education: A Mixed-Meta Synthesis of Academic Achievement and Qualitative Learning Outcomes

**DOI:** 10.3390/jintelligence14100256

**Published:** 2026-10-12

**Authors:** Ahmet Akif Erbaş, Nagihan Evci, Ceylan Akcan, Emrullah Akcan, Erkan Yeşiltaş

**Affiliations:** 1 Ahmet Erdoğan School of Health Services, Zonguldak Bülent Ecevit University, Zonguldak 67100, Türkiye; 2 Faculty of Education, Sivas Cumhuriyet University, Sivas 58140, Türkiye; evcinagihan@gmail.com (N.E.); ceylanakcan87@gmail.com (C.A.); erkanyesiltas@gmail.com (E.Y.); 3 Nizip Faculty of Education, Gaziantep University, Gaziantep 27310, Türkiye; emrullahakcan@gmail.com

**Keywords:** geographic information systems, social studies education, spatial thinking, meta-analysis, meta-thematic analysis

## Abstract

This study aimed to synthesize quantitative and qualitative evidence regarding the use of Geographic Information Systems (GIS) in social studies education through a mixed-methods meta-analysis that combines meta-analysis and meta-thematic analysis. As part of the quantitative synthesis, seventeen experimental and quasi-experimental studies were thematically analyzed, while seventeen qualitative studies were thematically analyzed as part of the qualitative synthesis. The results of the meta-analysis, which employed a random-effects model, indicated that the use of GIS has a significant positive effect on participants in the intervention groups’ academic achievement (Hedges’ g = 1.021). Moderator analyses revealed that sample size (Q = 0.382, *p* = .826), grade level (Q = 3.082, *p* = .214), duration of implementation (Q = 1.309, *p* = .520), and publication type (Q = 2.977, *p* = .226) did not result in statistically significant differences in effect sizes. The meta-thematic analysis identified six themes: higher-order thinking and cognitive skills, the learning process and environment, map literacy and spatial skills, awareness, emotional and motivational contributions, and technological and practical challenges. Qualitative findings indicate that, in the studies examined, the use of GIS was associated with positive contributions to participants in the intervention groups’ cognitive processes, spatial thinking and reasoning, learning experiences, awareness, and motivation. However, these qualitative findings were not interpreted as evidence of objective improvement in participant’ cognitive or affective characteristics. Similarly, no direct link or causal mechanism at the study level was tested between qualitative themes and quantitative effect sizes related to academic achievement. Therefore, the findings suggest that quantitative evidence of the positive impact of GIS use on academic achievement and the learning experiences and perceived benefits reported in qualitative studies provide complementary, yet non-causally linked, evidence.

## 1. Introduction

Over the past quarter-century, information and communication technologies have reshaped education systems worldwide, changing both the tools teachers use and the assumptions governing how knowledge is taught and learned. As economies place a growing premium on lifelong learning, the transmission of fixed content through conventional means has become increasingly difficult to justify (Halász & Michel, 2011). Comparative evidence reinforces the point: OECD assessments show that students who work with digital inquiry tools outperform peers taught conventionally on complex reasoning tasks (OECD, 2021). Equipping schools to use these technologies is therefore no longer a matter of institutional preference but a structural requirement, one that depends heavily on the digital competence of teachers and students (Guitert et al., 2021). Within this wider shift, spatially oriented technologies have drawn particular attention, since spatial reasoning is increasingly recognized as a foundational competency underpinning achievements across the STEM disciplines and the social sciences (Newcombe, 2017; National Research Council, 2006).

Technological change has done more than digitize resources; it has reoriented pedagogical thinking away from teacher-center transmission toward student-center, constructivist practice. The passive learner who reproduces information has given way to an active learner who questions, analyses, and reconstructs knowledge in meaningful contexts. Constructivist theory holds that durable understanding develops when learners engage with authentic problems and representational tools rather than decontextualised content (Jonassen et al., 1999), and recent classroom research continues to confirm that active, inquiry-oriented designs deepen learning and engagement (Galindo-Melero et al., 2024). These demands are sharpest in domains where knowledge must be made spatial, visualized, and tied to real-world phenomena, conditions that point directly toward geospatial technologies such as Geographic Information Systems (GIS) (Bednarz, 2004).

Social studies is a multidisciplinary field drawing on history, geography, economics, sociology, anthropology, and psychology to give learners the knowledge, skills, and values needed for informed civic participation (Ünal & Sarı, 2012). Its central aim is to cultivate responsible, democratic citizens who understand the communities they inhabit. Türkiye’s 2005 curriculum reform formally adopted constructivism as the foundation of social studies, placing inquiry and knowledge construction at the center of the program. Yet research across several national contexts shows that geography-related content and spatial reasoning remain among the hardest skills to develop when teaching relies on printed maps and teacher-led exposition. Jo and Bednarz (2009), analyzing geography textbook questions, found that even materials with explicit spatial content rarely demand genuine spatial reasoning, a shortfall that persists regardless of curricular intent. Evidence from Türkiye is similar: Elbay (2020) reported that the 2018 Social Studies Curriculum and its textbooks offer few opportunities for spatial thinking across all three middle-school grades, naming it as a goal without supplying the instructional scaffolding to reach it. GIS has emerged as the technology best matched to the constructivist aims of social studies, precisely because it turns students from passive map readers into active spatial investigators (Ünal & Sarı, 2012).

GIS can be defined as an integrated system of hardware, software, data, methods, and expertise for collecting, storing, analyzing, and displaying spatial data about the physical and human features of the Earth’s surface (Longley et al., 2015). What sets it apart from conventional maps is its capacity for geographic analysis: overlaying multiple data layers to generate knowledge that no single dataset could reveal. In schools, a useful distinction separates education about GIS, technical training in software typical of university programs, from education with GIS, the mode relevant to primary and secondary settings, where the technology serves as a vehicle for inquiry, not an end in itself (Bednarz, 2004). GIS entered classrooms from the 1990s as hardware costs fell, with early integration efforts in the United States, the United Kingdom, and several European countries. Kerski et al. (2013) later found that even where curricula mandate GIS, classroom use remained shallow because institutional conditions seldom matched policy ambition.

A substantial body of research documents the benefits of GIS-integrated instruction, including gains in achievement, motivation, spatial thinking and map literacy, and higher-order inquiry skills (Ünal & Sarı, 2012). Experimental work reinforces the pattern: Yang et al. (2024) found that GIS-supported instruction on air pollution produced higher post-test scores than conventional teaching in Chinese secondary schools; Bikar et al. (2022) reported greater intrinsic motivation among underachieving Malaysian students; and Bodzin (2011) documented gains in spatial reasoning among urban middle-schoolers. The National Research Council (2006) identified GIS as a leading support for spatial thinking across K-12, and Favier and Schee (2014) showed that GIS-supported inquiry strengthened Dutch students’ relational geographic thinking. At an aggregate level, Ma et al. (2023) reported a medium positive effect of geospatial technologies on learning, moderated by country and education level, while Rubino-Hare et al. (2024) found that a nationally scaled inquiry program raised student interest in STEM careers, with teacher professional development quality as a key moderator.

Despite this evidence, GIS has not reached the classroom integration its advocates expected, in Türkiye or in better-resourced systems. Reviewing 34 studies, Bernhäuserová et al. (2022) identified 68 barriers and found teacher-related factors, namely confidence, pedagogical content knowledge, and time, to be the most frequently cited. Technical and infrastructural gaps, the scarcity of curriculum-aligned datasets, and weak teacher preparation compound the problem. In Türkiye, teacher education has not historically treated GIS as a substantive, practice-based subject, so many graduates barely encounter it. This aligns with the Technological Pedagogical Content Knowledge (TPACK) framework, which holds that effective integration requires combining technical skill with disciplinary content and sound pedagogy (Mishra & Koehler, 2006), a profile that competence frameworks such as DigCompEdu have sought to specify and develop (Caena & Redecker, 2019). Schulze’s (2021) review of 26 studies further showed that the field’s methodological quality is uneven and that the conditions under which GIS produces its strongest effects remain poorly understood.

International evidence on the educational effectiveness of Geographic Information Systems (GIS) has grown considerably; however, the findings remain heterogeneous across educational contexts, grade levels, subject areas, implementation characteristics, and study designs. Although experimental and quasi-experimental studies have generally reported positive effects of GIS-based instruction, the magnitude and consistency of these effects vary across studies, suggesting that the effectiveness of GIS may depend on contextual and methodological characteristics. Evidence from Türkiye remains comparatively limited, and the findings of individual studies have not yet been systematically integrated with the broader international evidence.

Recent research has highlighted the need for a more comprehensive synthesis of GIS effectiveness across different educational and cultural contexts. For example, Bondarenko (2025), in a synthesis of 257 studies published between 2010 and 2024, emphasized that evidence concerning the effectiveness of GIS across subject areas, cultural settings, and learner characteristics remains insufficient. This limitation is particularly relevant for educational systems such as Türkiye, where GIS-based instruction has been examined in a relatively limited number of studies and where findings from individual investigations may not provide sufficient evidence regarding the overall magnitude and consistency of GIS-related learning effects.

Accordingly, the present study addresses the need to systematically synthesize the available empirical evidence on the effectiveness of GIS-supported instruction. Rather than examining the effectiveness of a single intervention or thematic unit, this study integrates findings across existing quantitative and qualitative research to estimate the overall effect of GIS-based instruction and to examine whether this effect varies according to relevant study and implementation characteristics. In particular, the synthesis considers potential sources of heterogeneity, including grade level, implementation duration, publication type, and other characteristics reported in the included studies. By integrating evidence across studies and examining potential moderators of effect size, the study aims to provide a more comprehensive understanding of when, where, and under what conditions GIS-supported instruction may be most effective. This approach also enables the evidence from Türkiye to be considered within a broader international research context and contributes to a more evidence-based understanding of GIS integration in educational settings.

Against this background, the paper synthesizes theoretical and empirical knowledge on GIS in social studies education, weighs the evidence, identifies the main barriers to implementation, and sets out the research agenda needed for GIS to become a mainstream tool in Turkish social studies classrooms. Drawing on Turkish and international scholarship alongside recent meta-analytic work (Ma et al., 2023; Schulze, 2021), it argues that GIS is not merely professional software but a pedagogically powerful learning technology that, used well, can engage learners, deepen spatial and disciplinary understanding, and build the higher-order thinking that citizenship now demands.

### Purpose and Significance of the Study

While international evidence regarding the use of Geographic Information Systems (GIS) in social studies education is steadily increasing, it is evident that the distribution of studies is not balanced across different educational systems, grade levels, subject areas, and implementation conditions. In Turkey, the limited number of studies on the effectiveness of GIS in social studies education necessitates an evaluation of existing findings within the context of different samples and implementation conditions. Furthermore, assessing the effectiveness of GIS use not only through quantitative indicators such as academic achievement but also in conjunction with participants in the intervention groups’ views and experiences regarding its application can contribute to a more comprehensive understanding of GIS’s educational potential. In this regard, the significance of the present study lies in its ability to integrate quantitative and qualitative evidence from various studies on the use of GIS in social studies within a single framework, thereby enabling a more systematic evaluation of the scattered findings in the literature. Within this scope, quantitative research findings were synthesized through meta-analysis, while qualitative research findings were synthesized through meta-thematic analysis; both types of evidence were evaluated together using a mixed-methods meta-approach. In addition, variables that could play a role in the effectiveness of GIS such as sample size, grade level, duration of implementation and type of publication were taken into account. Thus, the study sought to determine not only whether GIS is effective, but also under which research and implementation conditions this effectiveness varies, and how participants in the intervention groups’ experience the GIS-supported learning process. In this context, the primary objective of the study is to comprehensively synthesize quantitative and qualitative research findings regarding the effectiveness of GIS use in social studies instruction through a mixed-methods approach. In line with this primary objective, the study sought to answer the following questions:

1. According to the studies examined in this meta-analysis,
  - What is the overall effect size of using GIS in social studies classes on students’ academic achievement?
  - Does the effect size of GIS use on academic achievement differ significantly according to the variables of sample size, grade level, duration of implementation (weeks), and publication type of publication?
2. In the studies examined within the scope of the meta-thematic analysis, what are the implications of GIS use for the learning process, based on participants in the intervention groups’ views and experiences?

## 2. Materials and Methods

This study examined the effectiveness of Geographic Information Systems (GIS) in social studies instruction within a mixed-meta methodological framework that integrates quantitative and qualitative findingsIn this document analysis-based approach, quantitative data were analyzed using Comprehensive Meta-Analysis (CMA), Version 2 (Biostat, Inc., Englewood, NJ, USA), while qualitative data were examined through meta-thematic analysis. This approach was implemented within the mixed-meta methodological framework described by Batdı (2020a). The mixed-meta method enables the integration of different data types to produce more comprehensive and multi-dimensional results; by bringing together independent studies conducted under varying conditions, it offers more reliable and generalizable conclusions (Batdı, 2017; Pluye & Hong, 2014; Thomas & Harden, 2008). Accordingly, meta-analysis was employed for the quantitative component and meta-thematic analysis for the qualitative component. The method priorities the inclusion of methodologically rigorous studies that contain sufficient data (Batdı, 2021) and aims to contribute deeper insights to the existing body of literature. The analytical stages of the mixed-meta process are described in detail below.

### 2.1. The Meta-Analysis Process

The quantitative dimension of this mixed-meta study drew on a meta-analytic approach. Within this framework, studies examining the effect of GIS use on participants in the intervention groups’ achievement in social studies were systematically reviewed. Meta-analysis involves the statistical re-evaluation of empirical studies conducted through different methodologies by aggregating them according to predetermined criteria (Siddaway et al., 2019). This approach allows the existing literature to be engaged in a more comprehensive and objective manner (Schmidt, 1992) while enabling more robust and generalizable conclusions to be reached through the integration of independent studies (L’Abbe et al., 1987).

It was examined whether the studies included in the quantitative analysis contained multiple reports derived from the same research. To this end, the publications were compared in terms of author(s), publication year, sample size, grade level, participant characteristics, and reported data. When multiple publications from the same research were identified, only one report was included in the analysis to prevent the same data from being entered into the meta-analysis more than once. As a result of this process, a doctoral dissertation was found to overlap with an article derived from the same research and reporting data from the same participants. Therefore, the dissertation was excluded from the analysis, and the article was retained. The main characteristics of the studies and the research information compared during this process are presented in the dataset used in the meta-analysis.

However, although the meta-analysis provides evidence of the pooled effect of GIS use on participants in the intervention groups’ achievement, it remains limited in explaining how and through which processes this effect emerges. Therefore, in the second phase, qualitative findings were synthesized through meta-thematic analysis to elucidate the pedagogical and experiential dimensions underlying the quantitative results.

### 2.2. Data Collection

To identify studies examining the use of GIS in social studies education and the impact of this use on participants in the intervention groups’ learning processes and academic achievement, a comprehensive literature review was conducted between 8 November 2025 and 25 December 2025. During the search process, the following resources were utilized: the ProQuest and Web of Science bibliographic databases; the academic publishing platforms Taylor & Francis, ScienceDirect, Springer Nature, and Wiley Online Library; the Google Scholar academic search engine; and the Higher Education Council National Thesis Center (YÖK) to access graduate theses completed in Turkey.

The search strategy was developed around two main groups of concepts representing the research topic: (i) Geographic Information Systems (GIS) and related technologies, and (ii) social studies education. In the English-language searches, terms such as “Geographic Information System,” “Geographic Information Systems,” “GIS,” “GIS application,” “social studies,” “social studies education,” “academic achievement,” and “student achievement” were used. In the Turkish literature review, the terms “Coğrafi Bilgi Sistemi,” “Coğrafi Bilgi Sistemleri,” “CBS,” “CBS uygulamaları,” “CBS eğitimi,” “sosyal bilgiler,” “sosyal bilgiler eğitimi,” and “akademik başarı” were used.

These terms were combined in various ways using Boolean operators such as AND and OR, in a manner appropriate for the search features of electronic resources. For example, in English searches, combinations such as (“Geographic Information System” OR GIS OR “GIS application” OR “GIS-based learning”) AND (“social studies” OR “social studies education”) were used. In Turkish searches, combinations such as (“Coğrafi Bilgi Sistem” OR CBS) AND (“sosyal bilgiler” OR “sosyal bilgiler eğitimi”) were used. To identify studies examining academic achievement and learning outcomes, terms related to outcome variables—such as “academic achievement,” “student achievement,” and “akademik başarı”—were added to these searches when necessary. The search syntax varied depending on the search interface and technical functionalities of each source.

Because source-specific search strings were not systematically recorded during the initial phase of the study, exact database-specific search strings that could not be verified were not retrospectively reconstructed or attributed to particular sources. Instead, the revised manuscript reports the sources searched, the search dates, the search terms used, the conceptual combinations, and the general Boolean search logic applied during the initial search process, with the aim of presenting the search procedure as transparently as possible.

Searches were conducted in the title, abstract, keyword, and subject/general search fields of the studies, taking into account the search interface and indexing features of each electronic resource. In this way, the search was not limited to studies containing specific keywords in their titles; the aim was also to identify studies related to the subject but indexed under different terms. Because the search features of the sources used varied terms, search queries and Boolean combinations were adapted to suit the technical specifications of each source.

The records identified through the screening process were compiled, and duplicate records were removed. The studies were then reviewed at the title and abstract levels; those deemed relevant to the study’s objective and the predefined inclusion criteria were selected for full-text evaluation. The suitability of the studies was assessed separately for quantitative meta-analysis and qualitative meta-thematic analysis. For the quantitative meta-analysis, studies were included that examined the use of GIS in social studies education, employed an experimental or quasi-experimental research design, included experimental and control or comparison groups, and provided sufficient statistical data to calculate the effect size on academic achievement. For the meta-thematic analysis, studies that addressed the use of GIS in social studies education and provided analyzable qualitative findings relevant to the research objectives were evaluated.

Two researchers conducted the assessments regarding the inclusion and exclusion of studies. In cases where discrepancies arose during the assessments, the relevant studies were re-examined, and a consensus was reached through discussion. No standardized quality assessment tool or formal risk-of-bias assessment was used during the study selection process; the eligibility of the studies was evaluated based on predefined inclusion and exclusion criteria.

**Figure 1:**
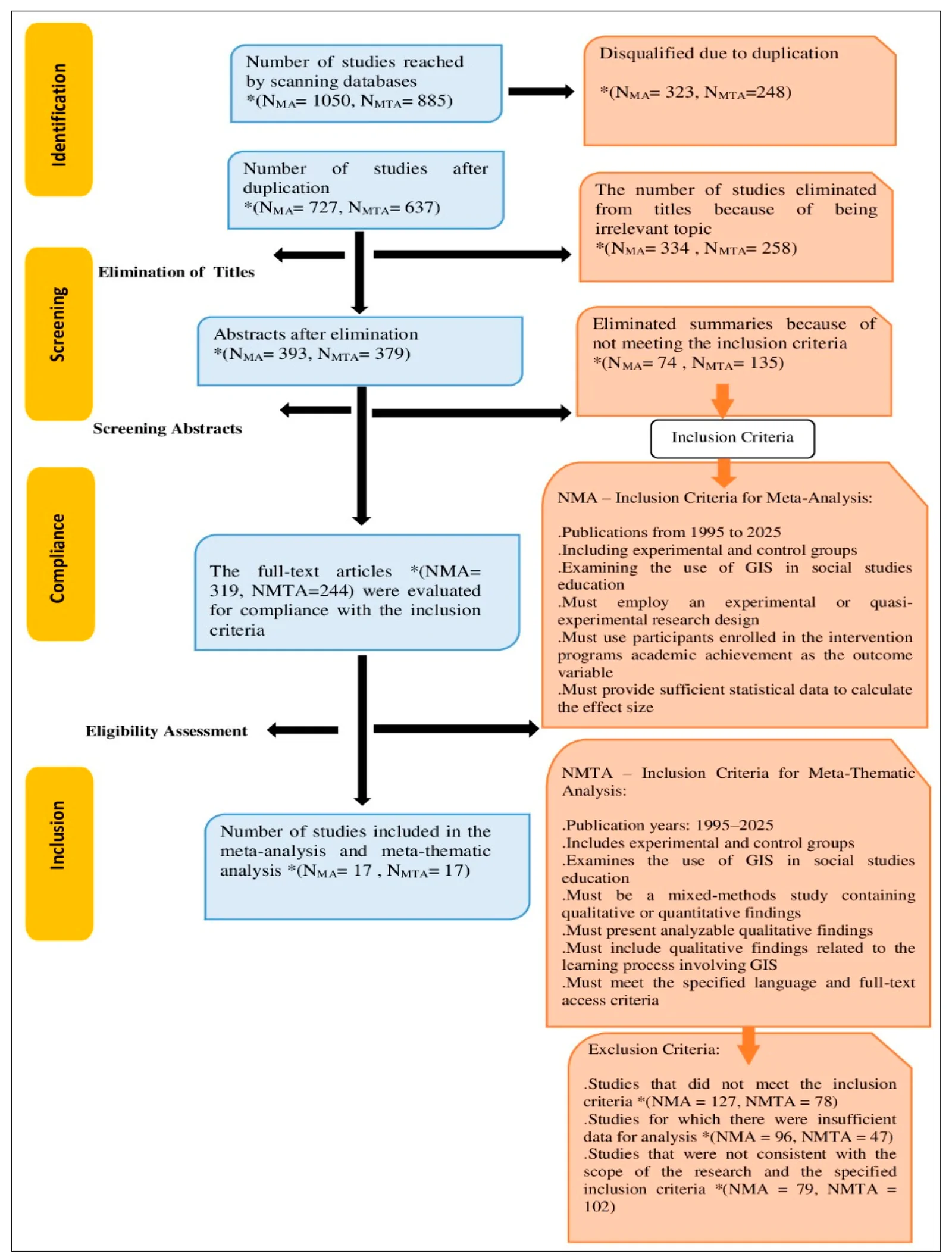
PRISMA flow diagram of studies included in the analysis (note: * NMA = meta-analysis; NMTA = studies included in the meta-thematic analysis scope).

In addition to the initial searches conducted in electronic sources, the reference lists of potentially relevant studies were reviewed retrospectively, and subsequent citations to those studies were also searched prospectively. The additional studies identified during this process were evaluated according to the same inclusion and exclusion criteria. Since the number of records obtained from each electronic source was not recorded separately during the screening process, exact record counts by source could not be reported retrospectively. However, after combining the records obtained from all sources, a total of 1050 records were identified; following the removal of duplicate records, the studies were evaluated separately for quantitative meta-analysis and qualitative meta-thematic analysis. The processes of identifying, screening, assessing the eligibility of, and including studies in the synthesis are illustrated in the flowchart presented in Figure 1. As a result, 17 studies were included in the meta-analysis, and 17 studies were included in the meta-thematic analysis. The inclusion and exclusion criteria are presented in Table 1.

The eligibility of studies to be included in the research within the scope of the mixed-methods meta-method was assessed by two researchers in accordance with the criteria established for quantitative meta-analysis and qualitative meta-thematic analysis. In the selection of studies, titles and abstracts were first reviewed, and studies deemed relevant to the study’s purpose and scope were selected for full-text evaluation. During the full-text review phase, the studies were evaluated methodologically and substantively based on the inclusion and exclusion criteria presented in Table 1. Duplicate records and studies that did not meet the criteria regarding the scope of the study, year of publication, language of publication, or research design were excluded.

Studies to be included in the quantitative meta-analysis were required to (a) examine the use of GIS in social studies education, (b) employ an experimental or quasi-experimental research design, (c) include an outcome variable related to participants in the intervention groups’ academic achievement, (d) meet the specified publication year and language criteria, and (e) provide sufficient statistical information to calculate the effect size. In particular, studies that did not adequately report data necessary for calculating the effect size—such as sample size, mean, and standard deviation—were excluded from the quantitative synthesis, even if they were appropriate in terms of subject matter and research design.

The studies to be included in the meta-thematic analysis were required to meet the following conditions: (a) they must address the use of GIS in social studies education; (b) they must meet the specified publication year and language criteria; (c) they must contain qualitative data; and (d) they must present analyzable qualitative findings regarding the impact of GIS use on the teaching–learning process. Studies containing qualitative findings—such as participants’ views, experiences, and perceptions regarding GIS use; GIS’s contributions to the learning process; and problems encountered during implementation—were evaluated within the scope of the meta-thematic analysis. Studies whose qualitative findings were not relevant to the research objective or that did not provide analyzable qualitative data were not included in the qualitative synthesis.

If a study met the inclusion criteria for both quantitative and qualitative components, it was evaluated within the scope of both syntheses. In contrast, studies that met the criteria for only the relevant component were included only in that component. For example, a study that did not provide sufficient quantitative data for calculating effect size but contained analyzable qualitative findings relevant to the research objective was excluded from the meta-analysis but included in the meta-thematic analysis.

Assessments regarding the inclusion or exclusion of studies were conducted by two researchers; when discrepancies arose between assessments, the relevant study was reviewed jointly, and a consensus was reached through discussion and agreement. However, a formal inter-rater reliability coefficient was not calculated during the initial study selection process, nor was a standardized quality assessment tool used. The eligibility of the studies was assessed based on the inclusion and exclusion criteria specified in Table 1. The number of studies excluded at each stage of the selection process and the reasons for exclusion are shown in the PRISMA flow diagram presented in Figure 1 (Page et al., 2021).

The PRISMA flow diagram presented in Figure 1 illustrates the processes of identifying, screening, assessing the eligibility of, and including studies in the synthesis for a meta-analysis (NMA) and a meta-thematic analysis (NMTA) conducted within the framework of a mixed-methods meta-methodology. The PRISMA flow diagram presented in Figure 1 illustrates the processes of identifying, searching for, assessing the eligibility of, and including studies in the synthesis for quantitative meta-analysis (NMA) and qualitative meta-thematic analysis (NMTA) conducted within the framework of a mixed-methods meta-methodology. As a result of searches conducted in databases and relevant sources, a total of 1050 records were identified for the meta-analysis (NMA) and 885 records for the meta-thematic analysis (NMTA). During the identification phase, duplicate records (NMA = 323, NMTA = 248) were removed; the remaining records (NMA = 727, NMTA = 637) were included in the screening process. During the title screening phase, studies determined to be irrelevant to the topic (NMA = 334, NMTA = 258) were excluded; the remaining records were then evaluated at the abstract level (NMA = 393, NMTA = 379). Records that did not meet the inclusion criteria during the abstract review (NMA = 74, NMTA = 135) were excluded, and the full-text review was then conducted.

During the full-text screening stage, 319 quantitative studies (NMA) and 244 qualitative studies (NMTA) were assessed according to the predefined inclusion and exclusion criteria. For the quantitative analysis, 127 studies were excluded because they did not meet the inclusion criteria, 96 because they did not provide sufficient statistical information to calculate effect sizes, and 79 because they fell outside the scope of the study. Among these 79 studies, a doctoral dissertation and an article derived from the same research were identified. A comparison of the two records showed that the dissertation and the article reported data from the same sample and research. To prevent the same data from being included twice in the quantitative synthesis, the dissertation was removed from the dataset, while the article was retained. This procedure did not result in any reclassification of the exclusion categories; the dissertation remained classified among the 79 studies excluded for being outside the scope of the study.

For the qualitative analysis, 78 studies were excluded for not meeting the inclusion criteria, 47 for not providing sufficient qualitative data for analysis, and 102 for falling outside the scope of the study. Ultimately, 17 quantitative studies (Table 2) that fully met the inclusion criteria were included in the meta-analysis (NMA), and 17 qualitative studies (Table 3) were included in the meta-thematic analysis (NMTA).

The study selection process was conducted by two researchers, and any differences in assessments regarding inclusion or exclusion were resolved through discussion and consensus. However, no formal inter-rater reliability coefficient was calculated during the study selection process, and no standardized quality assessment tool was used. The figures presented in Figure 1 reflect the eligibility assessments conducted separately for the quantitative meta-analysis and the qualitative meta-thematic analysis.

### 2.3. Data Analysis

Data analysis was conducted using the CMA 2.0 software. In the meta-analysis process, Hedges’ g was used as the effect size measure to determine the magnitude of the relationships between variables, particularly because it incorporates a correction factor that reduces bias that may arise from small sample sizes (Borenstein et al., 2013; Batdı, 2024). Effect sizes were calculated using CMA 2.0 based on the post-test mean scores, standard deviations, and sample sizes of the experimental and control groups (g = d × J). Accordingly, pretest scores were not taken into account in the effect size calculations. To ensure the independence of the effect sizes, only one effect size was selected from each study, and studies involving one experimental group and one control group were included in the analyses. Positive Hedges’ g values indicate an effect in favor of the experimental group. Corrections to reduce small-sample bias were automatically applied by the CMA 2.0 software. The classification by Thalheimer and Cook (2002) was used as the basis for interpreting the effect sizes; according to this classification, values between −0.15 and 0.15 are negligible, values between 0.15 and 0.40 are small, values between 0.40 and 0.75 are moderate, values between 0.75 and 1.10 are large, values between 1.10 and 1.45 are very large, and values above 1.45 are considered to represent an excellent effect. To arrive at more generalizable results while accounting for potential differences and heterogeneity among the studies, the Random Effects Model was used. Finally, a funnel plot and Fail-Safe N analysis were utilized to assess the reliability of the findings and potential publication bias.

### 2.4. Moderator Analysis

GIS use, and the I^2^ value was found to be 88.172. Considering that I^2^ values of 75% and above indicate a high level of heterogeneity (Cooper et al., 2009), it was determined that it would be appropriate to examine the findings in terms of various variables. Accordingly, sample size, grade level, duration of implementation and publication type of publication were considered as moderator variables, and subgroup analyses were conducted for each variable.

### 2.5. The Meta-Thematic Analysis Process

As the second stage of this study, meta-thematic analysis was adopted to complement the quantitative findings. This approach, which is grounded in the re-analysis of raw qualitative findings from existing studies in the literature to generate codes and themes (Batdı, 2020b), facilitated the integration of the quantitative and qualitative data. In doing so, it enabled a more systematic and in-depth synthesis of the research domain. This type of analysis aims to evaluate qualitative studies from both national and international contexts within a common framework through document review. In the process, the findings of different studies are recoded individually and synthesized into overlapping or divergent thematic structures. Rather than merely combining data, this approach re-interprets raw findings to identify shared theoretical ground, thereby offering a depth of understanding that transcends individual studies (Batdı, 2019). The concurrent use of meta-analysis and meta-thematic analysis, consistent with the nature of the mixed-method design, allowed the findings to be examined comparatively and thereby enhanced the reliability of the conclusions.

### 2.6. Data Collection and Analysis

Qualitative studies on GIS use in social studies were collected through document analysis, a method grounded in the systematic examination of written sources (Karasar, 2005; Özkan, 2019). Study selection was carried out following the four-stage PRISMA elimination process and in accordance with differentiation criteria including topical relevance, inclusion of qualitative data, and accessibility of full text (Batdı & Anıl, 2021). In accordance with the specified criteria, 17 studies were included in the analysis. The included studies are presented in Table 3.

Data obtained from the selected studies were subjected to content analysis, a procedure that enables the systematic coding and categorization of textual data according to defined rules (Büyüköztürk et al., 2016). Within this thematically structured framework, shaped by the researcher in line with the existing literature and theoretical foundations, direct participant quotations were included to enhance transparency and validity (Merriam, 2009). Qualitative data were meticulously analyzed at the main theme, subtheme, and code levels using computer-assisted data analysis software. To ensure traceability and transparency in the study, a unique code symbol (M1, T1, etc.) was assigned to each study, and the data was organized into a systematic structure. Accordingly, the six main themes identified were categorized and symbolized as “A1, A2, ….”

### 2.7. Coding Process

Coding, one of the fundamental stages of meta-thematic analysis, ensures that data are conceptualized and transformed into meaningful structures (Merriam, 2009; Batdı, 2023). In this process, primary participant quotes obtained from 17 studies (M1, T1, etc.) were systematically transcribed into a coding table. To ensure that the evidence was represented evenly across sources and to prevent a limited number of studies from contributing disproportionately to the themes, a comprehensive table was created showing the study characteristics (participants, settings, and methods) and each study’s relative contribution to the identified themes; this is presented in Table 3. Subsequently, the two researchers coded the participant data and the analytical themes derived from them independently of one another. Disagreements between the coders were resolved through consultation with subject matter experts, as well as through iterative discussions and consensus-building among the coders. In the reliability analysis conducted to determine the level of agreement among the coders, Cohen’s kappa coefficient was found to range between 0.82 and 0.88. Cohen’s kappa coefficient was used to determine the actual level of agreement in the coding process because it accounts for the portion of agreement that could arise by chance (Cohen, 1960). The coefficients obtained indicate a high level of agreement among the coders (Appendix A). The finalized codes were grouped under six main themes—“Higher-Order Thinking and Cognitive Skill Development,” “The Learning Process and Contributions to the Learning Environment,” “Map Literacy and the Development of Spatial Skills,” “Awareness,” “Emotional and Motivational Benefits,” and “Issues Relating to Technology and Implementation”—and presented visually.

### 2.8. Validity and Reliability

A comprehensive validity and reliability process was conducted to ensure the scientific quality, accuracy, and consistency of the study (Arslan, 2022). To ensure external validity (generalizability), all methodological stages of the study were reported transparently (Tong et al., 2012); data falling outside the scope of the study were excluded from the analysis; and direct quotations from the raw data were included in the findings section (Silverman, 1993/2018). The sources included in the analysis were systematically coded as articles (M) and theses (T). To ensure the reliability of the study, particularly in terms of inter-coder reliability and confirmability, independent coding and continuous comparison techniques were employed (Lincoln & Guba, 1985). Inter-coder agreement was assessed using two complementary approaches. First, Cohen’s kappa coefficient was calculated to account for any agreement that might arise by chance. The kappa coefficients ranged from 0.82 to 0.88, indicating a high level of agreement among the coders (Appendix A, Supplementary Materials). Second, the direct percentage agreement between the coders’ independent classifications was calculated using the formula Reliability = Agreement/(Agreement + Disagreement) proposed by Miles and Huberman (1994), and the total agreement rate was found to be 92.27%. Therefore, Cohen’s kappa coefficients and the Miles and Huberman percentage agreement rate were considered as two distinct measures derived from the same independent coding process that complementarily assess the agreement among coders.

## 3. Results

This section presents the findings obtained from the meta-analysis and meta-thematic analyses conducted within the scope of the study. Quantitative data were analyzed through meta-analysis, and qualitative data through the meta-thematic analysis approach.

### 3.1. Findings from the Meta-Analysis

Under this heading, the meta-analysis findings are presented; effect size results are reported first, followed by the moderator analysis findings.

#### Effect Size Results for Academic Achievement

**Figure 2:**
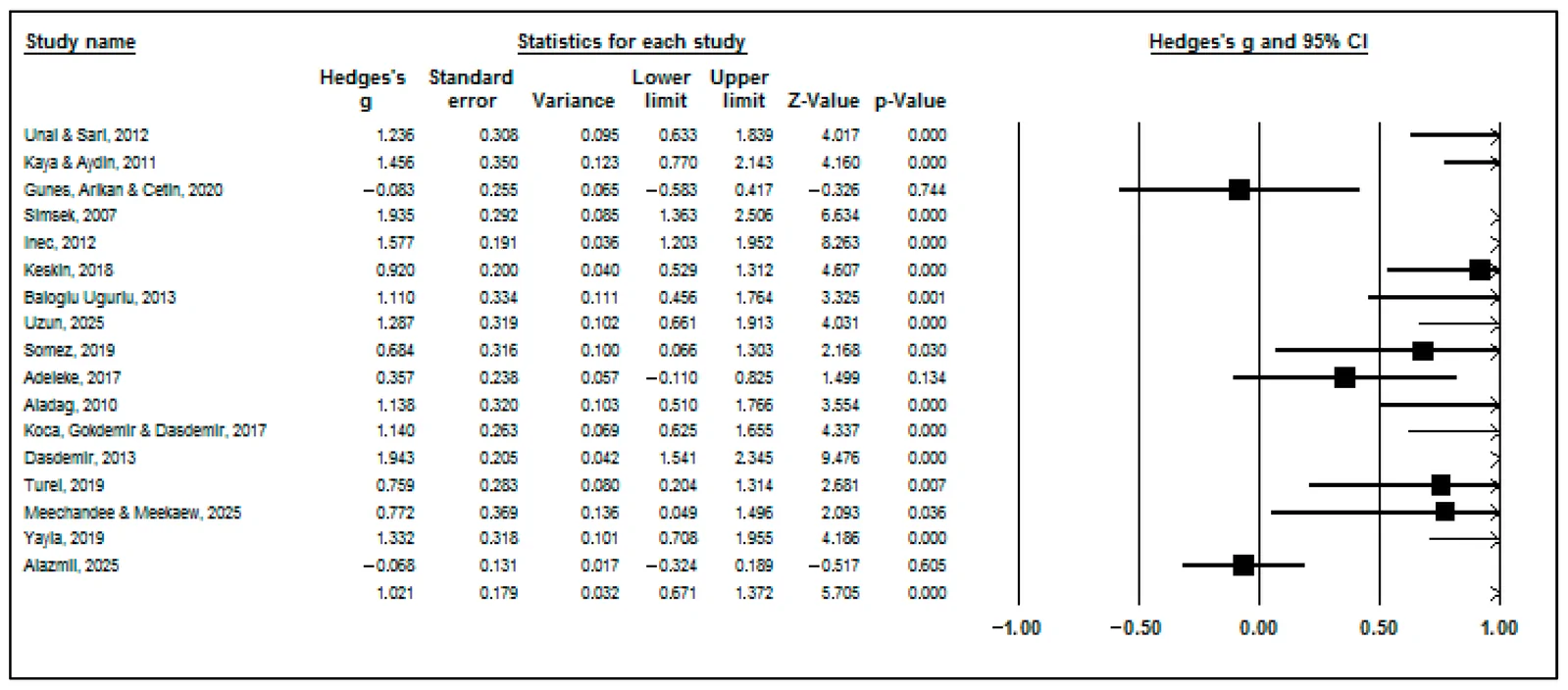
Forest Plot of the Effect Sizes Included in the Meta-Analysis. Note. Squares indicate effect sizes, horizontal lines indicate 95% confidence intervals, and arrows indicate confidence intervals extending beyond the displayed scale (Ünal & Sarı, 2012; Kaya & Aydın, 2011; Güneş et al., 2020; Şimşek, 2007; İneç, 2012; Keskin, 2018; Baloğlu Uğurlu, 2013; Uzun, 2025; Sönmez, 2019; Adeleke, 2017; Aladağ, 2010; Koca et al., 2017; Daşdemir, 2013; Türel, 2019; Meechandee & Meekaew, 2025; Yayla, 2019; Alazmi, 2025).

A forest plot showing the effect sizes of the studies included in the review is presented in Figure 2. The vast majority of the studies were found to show a significant effect in favor of the experimental group. However, two studies (Güneş et al., 2020; Alazmi, 2025) were found to have a significant effect size in favor of the control group. It was determined that the highest effect size value obtained in the study was 1.943 (Daşdemir, 2013), while the smallest effect size value was −0.083 (Güneş et al., 2020).

In the meta-analysis conducted using 17 studies, the Q statistic and I^2^ values were examined to assess heterogeneity (Table 4). The analysis yielded a Q value of 135.268 (*p* < .001) and an I^2^ value of 88.172%. Based on these values, a heterogeneous structure was identified. Due to the heterogeneity, a random-effects model was analyzed, and the effect size was found to be 1.021 (95% CI: 0.671–1.372), which was statistically significant (*p* < .001). However, a high degree of heterogeneity was observed among the effect sizes of the studies included in the meta-analysis (Q(16) = 135.268, *p* < .001, I^2^ = 88.172%, τ^2^ = 0.467). The 95% prediction interval ranged from −0.51 to 2.55. This result indicates that, although the overall effect is positive, due to the high heterogeneity among the studies, the true effect size in future studies of a similar nature could vary significantly and may fall within a range extending from negative to positive. These findings suggest that the differences in effect sizes observed across studies cannot be attributed solely to sampling error but may also stem from differences in the characteristics of the studies. Finally, a funnel plot was created to visually assess the studies included in the meta-analysis for potential publication bias, and the resulting plot is presented in Figure 2.

**Figure 3:**
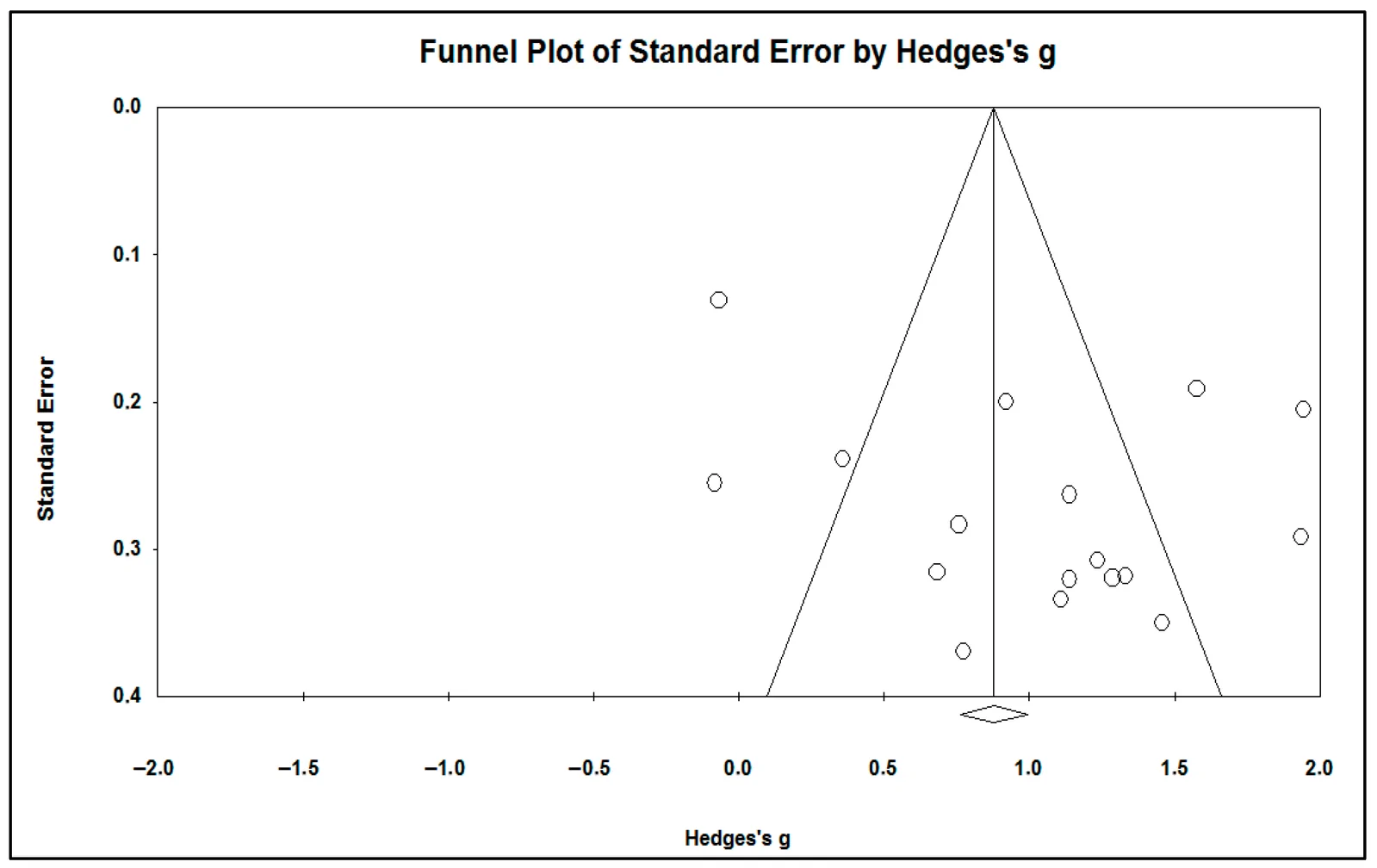
Funnel Plot. Note. Each point represents an individual study, and the vertical line indicates the overall effect size.

Figure 3 presents the funnel plot generated using CMA software. This figure provides a visual summary of the meta-analytic findings and allows for an assessment of potential publication bias (Cooper et al., 2009). A relatively symmetric distribution of effect sizes within the funnel boundaries may indicate a lower likelihood of publication bias, whereas substantial asymmetry may suggest the possibility of bias (Sarı & Şaşmaz Ören, 2020). Examination of Figure 3 shows that the studies generally exhibit a relatively symmetrical distribution, with no pronounced asymmetry observed. Studies to the right of the midline represent results favoring the experimental group, whereas those to the left represent results favoring the control group. The inclusion of a significant number of thesis studies in the sample and the incorporation of gray literature into the analysis may have helped reduce the risk of publication bias that could arise from a sample based solely on published studies. However, the inclusion of gray literature alone does not completely eliminate the possibility of publication bias. Therefore, the Fail-Safe N and the regression test developed by Egger et al. (1997) were applied to assess the studies included in the meta-analysis for publication bias. The Fail-Safe N value, calculated according to Rosenthal’s (1979) approach, was found to be 1056. This value indicates that 1056 additional studies with an average effect size of zero would be required to reduce the observed meta-analytic effect to a non-significant level. Given that 17 studies met the inclusion criteria, the substantially larger Fail-Safe N value indicates that a considerable number of additional unpublished or omitted studies with null findings would be required to reduce the observed pooled effect to a non-significant level.

Furthermore, Egger’s regression test was conducted to statistically assess the potential asymmetry observed in the funnel plot. The analysis yielded a *p*-value of .078. Since this value exceeds the .05 significance level, there was insufficient evidence to conclude that the asymmetry in the funnel plot was statistically significant. Taken together, the relatively symmetrical distribution observed in the funnel plot, the non-significant result of Egger’s regression test (*p* = .078), and the high Fail-Safe N value (N = 1056) indicate that there is no statistically significant evidence of substantial publication bias in the current meta-analysis. Nevertheless, these findings should not be interpreted as definitive evidence that publication bias is absent. In particular, given the limited number of studies included in the meta-analysis, the possibility of publication bias cannot be completely ruled out.

Although the funnel plot and Egger’s regression test did not provide statistically significant evidence of publication bias, a trim-and-fill sensitivity analysis was conducted to examine the robustness of the pooled effect to potential small-study effects. The analysis estimated four potentially missing studies and reduced the pooled Hedges’ g from 1.021 to 0.795. The adjusted effect remained statistically significant, indicating that the overall positive effect was not eliminated under this sensitivity analysis.

The random-effects meta-analysis revealed a large and positive effect of GIS use on academic achievement (g = 1.021). However, the reduction in the effect size from 1.021 to 0.795 following the trim-and-fill sensitivity analysis indicates that the overall effect estimate is somewhat sensitive to potential small-study effects. Nevertheless, the fact that the adjusted effect size remained statistically significant suggests that the positive effect of GIS use on academic achievement was not eliminated within the scope of this sensitivity analysis. Therefore, the overall effect size should be interpreted with caution, taking into account the high level of heterogeneity and the potential presence of small-study effects.

### 3.2. Moderator Analysis Results for Academic Achievement

Due to heterogeneity, the studies were subjected to moderator analyses with respect to the variables of sample size, grade level, duration of implementation and publication type. The results of the subgroup analyses conducted accordingly are presented in Table 5.

The moderator analyses presented in Table 5 indicate that none of the four moderator variables examined—sample size, grade level, intervention duration, and publication type—resulted in statistically significant differences in effect sizes. With regard to sample size, the difference between the groups was not statistically significant (Q = 0.382, *p* = .826). Similarly, grade level did not have a statistically significant moderating effect on the effect sizes (Q = 3.082, *p* = .214). Intervention duration also did not result in a statistically significant difference in effect sizes (Q = 1.309, *p* = .520). Likewise, publication type did not have a statistically significant moderating effect on the effect sizes (Q = 2.977, *p* = .226).

### 3.3. Findings from the Meta-Thematic Analysis

This section presents the findings structured through meta-thematic analysis. The qualitative data obtained during the analytical process were categorized under six main themes: “Higher-Order Thinking and Cognitive Skill Development “The Learning Process and Contributions to the Learning Environment”, “Map Literacy and the development of spatial skills”, “Awareness”, “Emotional and Motivational Benefits” and “Issues Relating to Technology and Implementation” The findings analyzed within this thematic framework are presented as a coherent analytical whole, supported by models developed to raise the level of abstraction and to render inter-data relationships concrete, alongside direct participant quotations and reference statements reflecting lived experiences. Accordingly, the findings pertaining to the first major theme to emerge during the analysis are detailed below.

**Figure 4:**
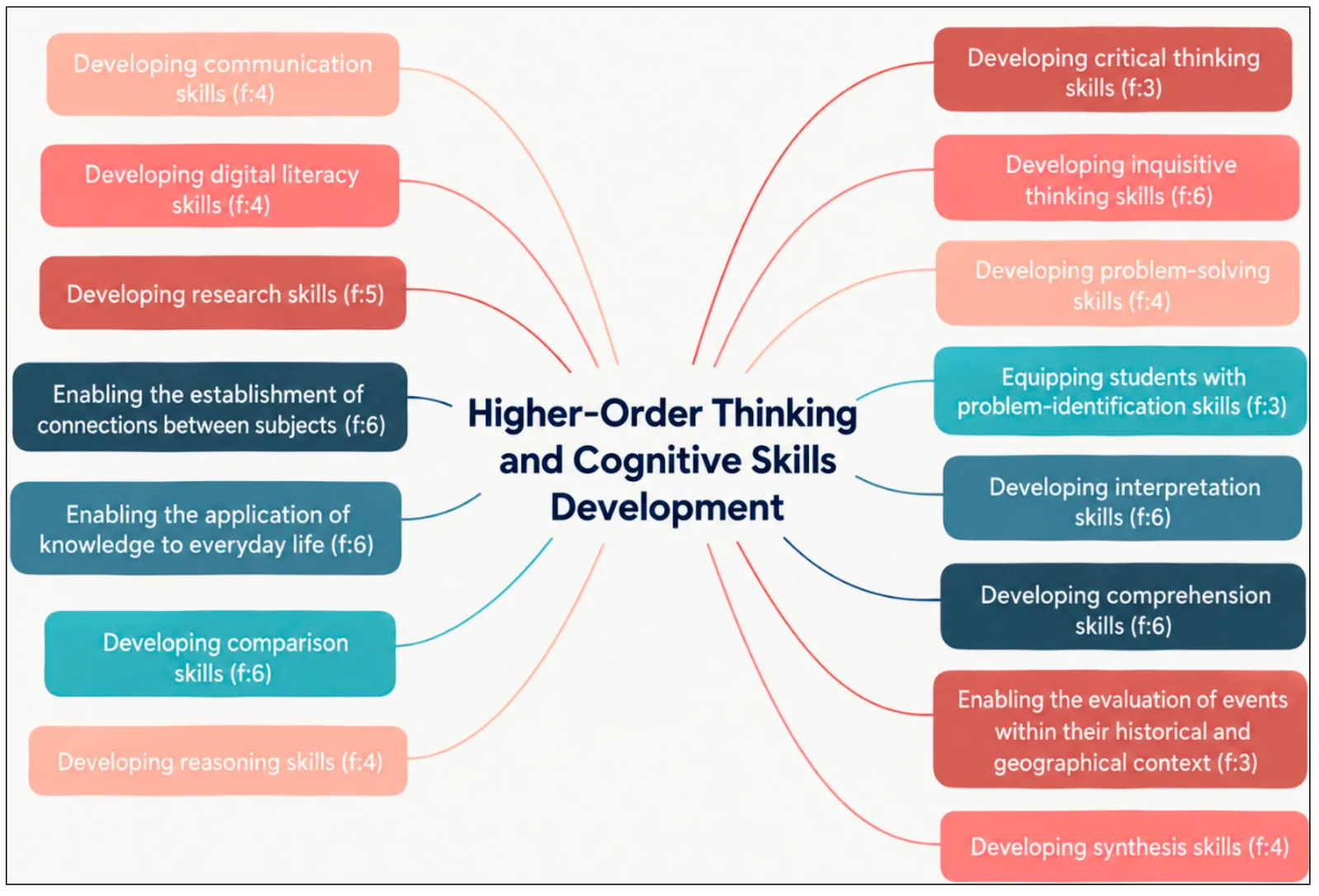
Higher-Order Thinking and Cognitive Skill Development. *Note. The colors of the boxes and their corresponding connecting lines are used only to visually distinguish the identified themes. The colors do not indicate different categories, levels, or relationships among the themes. The abbreviation “f” indicates the frequency of the corresponding theme.*

According to Figure 4, the theme of “Higher-Order Thinking and Cognitive Skill Development” is characterized by codes such as the development of critical thinking, inquisitive thinking skills, problem solving, problem identification, interpretation, comprehension, comparison, synthesis, research, reasoning skills and communication skills, as well as the acquisition of digital literacy skills. Among these, developing inquisitive thinking skills (f = 6), developing interpretation skills (f = 6), developing comparison skills (f = 6), developing comprehension skills (f = 6), enabling the application of knowledge to everyday life (f = 6), and enabling the establishment of connections between subjects (f = 6) were the most frequently identified codes. In contrast, developing critical thinking skills (f = 3), equipping students with problem-identification skills (f = 3), and enabling the evaluation of events within their historical and geographical context (f = 3) were the least frequently identified codes. In addition, codes related to developing the ability to evaluate events within their historical and geographical context, application to everyday life, and cross-topical connections were also prominent. Examining the participant reference statements obtained in the study, “*I can find out and search information about that place*, *and then I can learn a lot instead of just going through the book and stuff*, *and then you can get a lot of pictures so you can understand…*” (T2, s. 92), “*While looking at which places have dense populations*, *we also reinforce our knowledge of cities’ locations.*” (T1, p. 122), “*Learning by seeing more through GIS was positive; that is why I learned better.*” “…*we learn clearer and more detailed information.*” (M5, p. 51) *“Thanks to satellites*, *people can now access much more information about the Earth.*” “*The use of Geographic Information Systems is beneficial.*” (M8, p. 73), “*I believe that my overall success in class is increased with GIS.*” (M2, p. 415). Participants in the intervention groups’ views such as these emphasize the impact of the process on learning. These findings suggest that GIS use in social studies lessons may support participants’ skill acquisition as well as Higher-Order Thinking and Cognitive Skill Development in a holistic manner.

**Figure 5:**
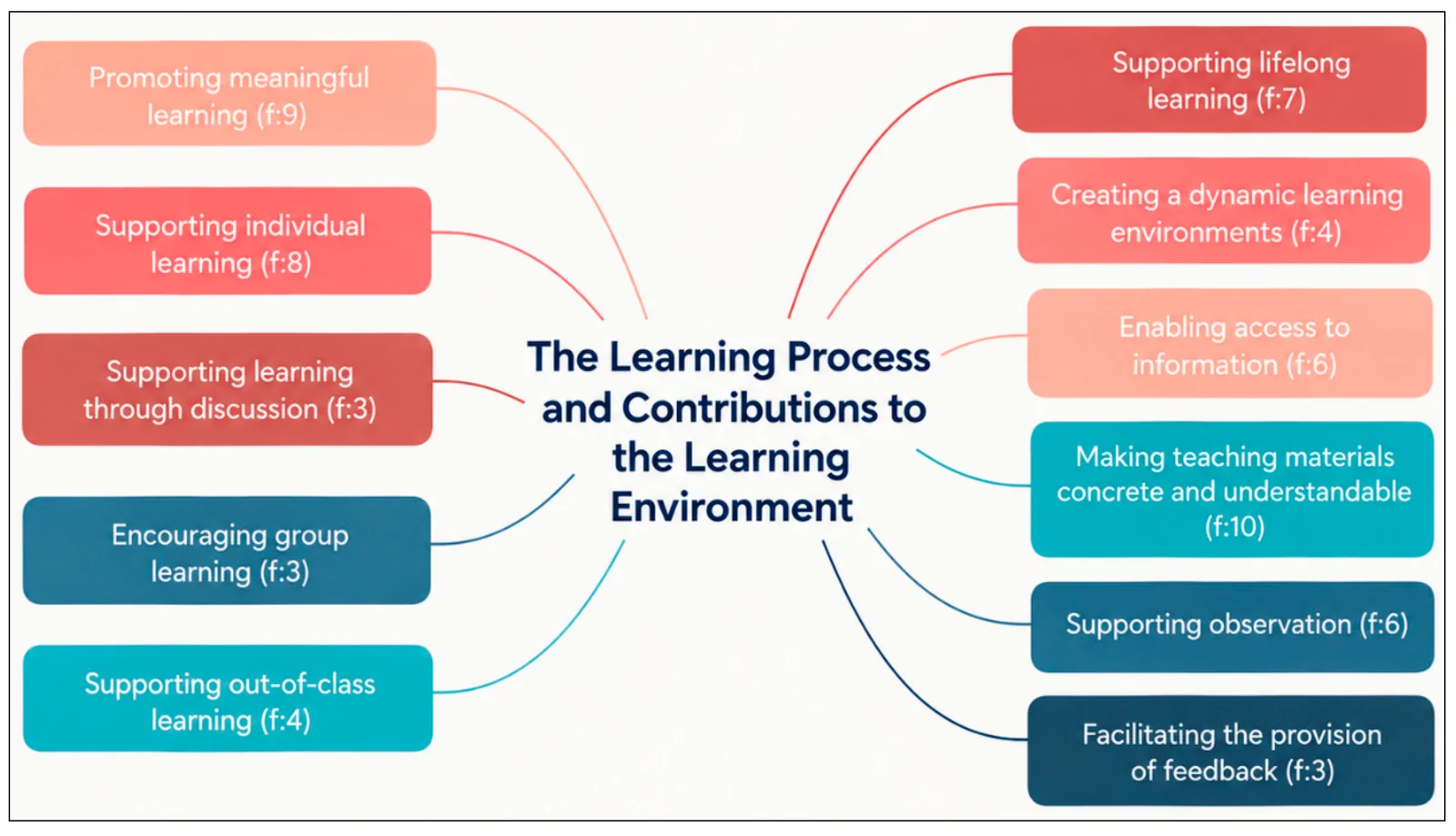
The Learning Process and Contributions to the Learning. Note. The colors of the boxes and their corresponding connecting lines are used only to visually distinguish the identified themes. The colors do not indicate different categories, levels, or relationships among the themes. The abbreviation “f” indicates the frequency of the corresponding theme.

According to Figure 5, the sub-themes and codes pertaining to the theme of “The Learning Process and Contributions to the Learning Environment” have been modelled. Under this main theme, the following codes are identified: promoting meaningful learning, supporting individual learning, supporting learning through discussion, encouraging group learning, supporting out-of-class learning, supporting lifelong learning, creating a dynamic learning environment, enabling access to information, making teaching materials concrete and understandable, supporting observation, and facilitating the provision of feedback. Among these, making teaching materials concrete and understandable (f = 10) was the most frequently identified code, followed by promoting meaningful learning (f = 9) and supporting individual learning (f = 8). In contrast, supporting learning through discussion (f = 3), encouraging group learning (f = 3), and facilitating the provision of feedback (f = 3) were the least frequently identified codes. Examining the reference statements, “*I can work on applications by myself on the computer.*” (M5, p. 50), draws attention to the dimension of individualized learning. Another study reported that, in response to the question *“How was your communication with your peers during the GIS applications?* participants gave responses such as *“We helped each other on topics we did not know”*, *“We communicated better”*, *“We also communicated with our teacher”* and *“We had the opportunity to discuss things.”* (T4, p. 165)—responses that reflect support for social interaction and peer learning.

**Figure 6:**
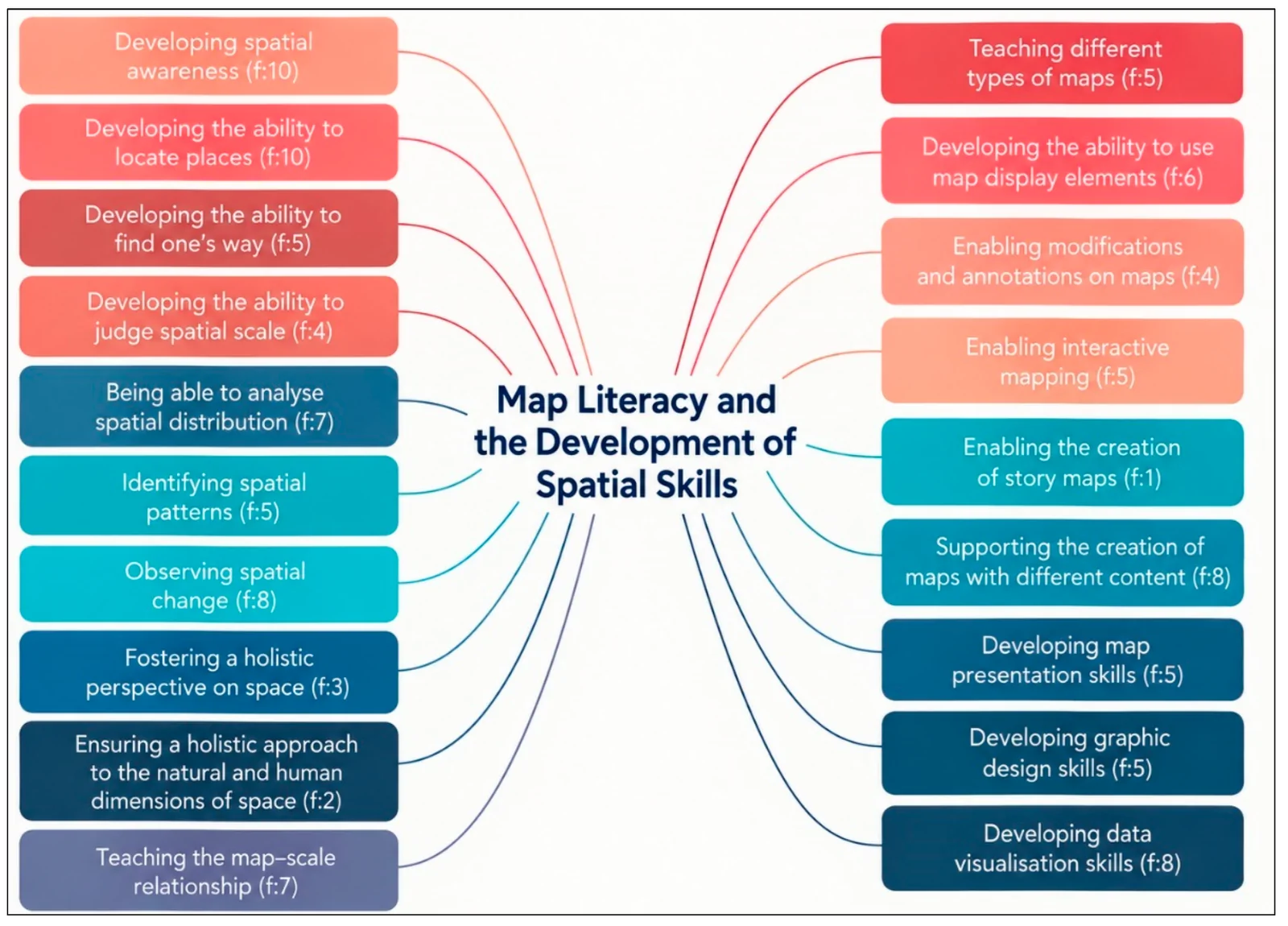
Map literacy and the development of spatial skills. Note. The colors of the boxes and their corresponding connecting lines are used only to visually distinguish the identified themes. The colors do not indicate different categories, levels, or relationships among the themes. The abbreviation “f” indicates the frequency of the corresponding theme.

According to Figure 6, the theme of “Map Literacy and the Development of Spatial Skills” is characterized by codes such as developing spatial awareness, developing the ability to locate places, developing the ability to find one’s way, developing the ability to judge spatial scale, being able to analyse spatial distribution, identifying spatial patterns, observing spatial change, fostering a holistic perspective on space, ensuring a holistic approach to the natural and human dimensions of space, teaching the map–scale relationship, teaching different types of maps, developing the ability to use map display elements, enabling modifications and annotations on maps, enabling interactive mapping, enabling the creation of story maps, supporting the creation of maps with different content, developing map presentation skills, developing graphic design skills, and developing data visualisation skills. Among these, developing spatial awareness (f = 10) and developing the ability to locate places (f = 10) were the most frequently identified codes, followed by observing spatial change (f = 8), supporting the creation of maps with different content (f = 8), and developing data visualisation skills (f = 8). In contrast, enabling the creation of story maps (f = 1) was the least frequently identified code, followed by ensuring a holistic approach to the natural and human dimensions of space (f = 2) and fostering a holistic perspective on space (f = 3).

Participant reference statements further corroborate the role of GIS in this process: *“thanks to GIS*, *my ability to read different maps has improved*, *and I’ve started interpreting them more easily. I believe that these skills will improve even further as I use GIS programs.”* (T8, p. 148), *“Map 1 shows Ankara*, *while Map 2 shows Istanbul.”*, *“Both maps are to the same scale and show the same location*, *but they have different features.”* (M9, p. 27), and *“Yes*, *I have learned about parallels and meridians very well.”* (T7. p. 77). These statements suggest that participants perceived GIS as contributing to this process.

**Figure 7:**
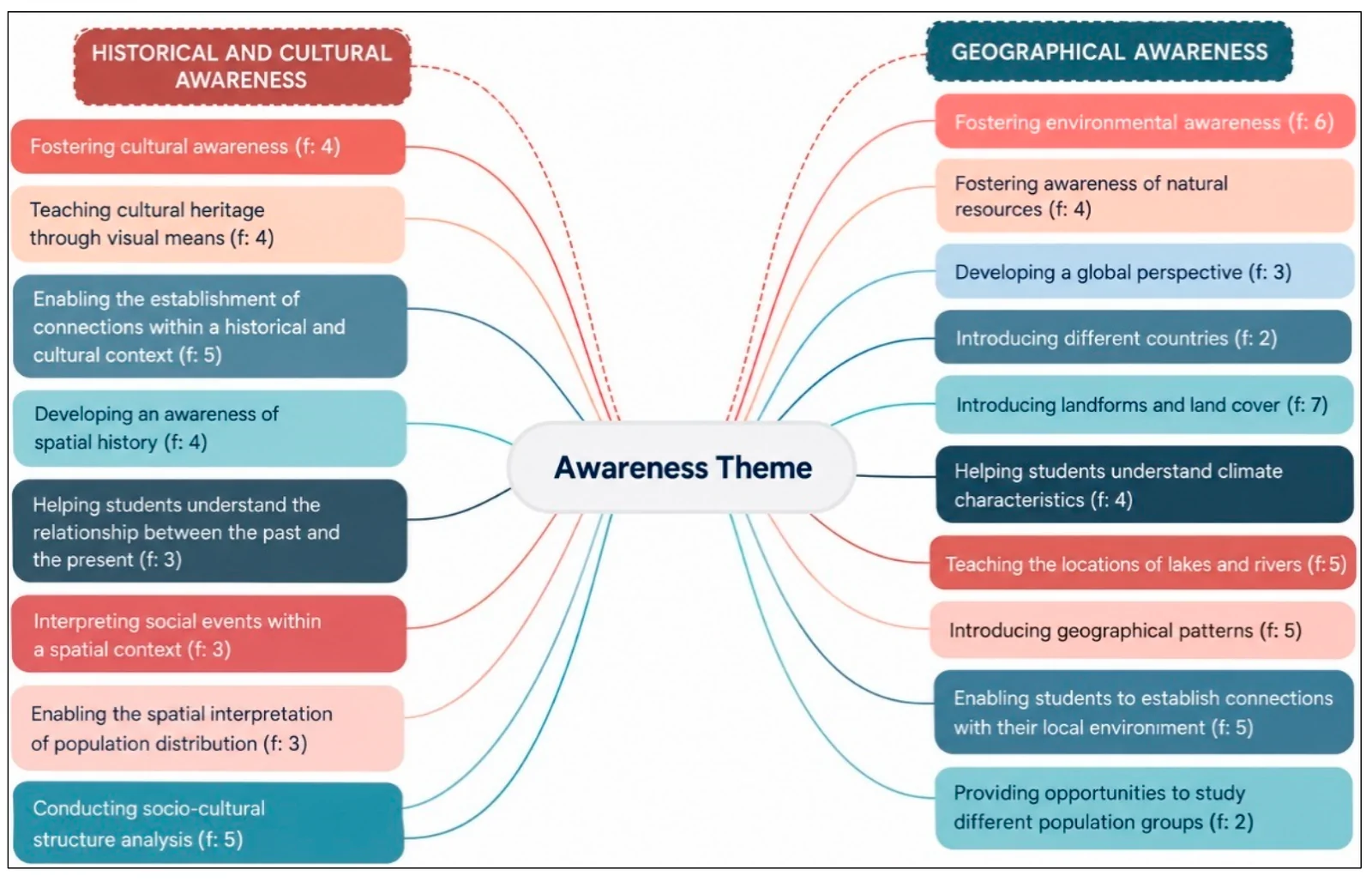
Awareness Theme. *Note. The colors of the boxes and their corresponding connecting lines are used only to visually distinguish the identified themes. The colors do not indicate different categories, levels, or relationships among the themes. The abbreviation “f” indicates the frequency of the corresponding theme.*

According to Figure 7, the “Geographical Awareness” sub-theme encompasses codes such as fostering environmental awareness, fostering awareness of natural resources, developing a global perspective, introducing different countries, introducing landforms and land cover, helping students understand climate characteristics, teaching the locations of lakes and rivers, introducing geographical patterns, enabling students to establish connections with their local environment, and providing opportunities to study different population groups. Among these, introducing landforms and land cover (f = 7) was the most frequently identified code, followed by fostering environmental awareness (f = 6). Teaching the locations of lakes and rivers (f = 5), introducing geographical patterns (f = 5), and enabling students to establish connections with their local environment (f = 5) were also frequently identified. In contrast, introducing different countries (f = 2) and providing opportunities to study different population groups (f = 2) were the least frequently identified codes. Participant views on this theme render concrete the impact of technology on geographic awareness. Example: *Bridgett and Dan focused on the opportunity provided by Story Maps to find different locations and directions. According to Bridgett*, *Story Maps can help “Find something like directions.” Dan agreed*, *stating “It’s a map*, *so it tells you your location.” Students also perceived a connection between Story Maps and learning geography. In fact*, *all the participants reported seeing the physical features of locations on Earth’s surface. For Bella*, *there is a connection between Story Maps and her life because it helps her understand what makes a place unique by letting her “know how it was built*, *and how it looks like from a map.”* (M1, p. 10). These statements indicate that participants in the intervention groups’ spatial experience and geographic awareness have expanded beyond abstract concepts into real-world connections. The connection between GIS and everyday life is further illustrated by “*With GIS*, *I have seen plains*, *mountains*, *and rivers I have never been to. When you see faraway places*, *it feels as if you were going there. Now I can see any place in the world”* (M3, p. 259) and *“Using GIS helped me see the whole picture and how the problem is distributed from a global lens.”* (M6, p. 17), *“Distances between countries and states could be calculated using the latitude and longitude.”* (T6, p. 75), *“Thanks to these*, *we can access information about different places*, *places we might never even see. In that respect*, *they are quite effective.”* (M7, p. 120), *“If someone asks us something about Türkiye*, *we will be able to answer them directly thanks to Google Earth”* (T2, p. 76). These data demonstrate that GIS use transforms theoretical geographical knowledge into practical and functional awareness.

**Figure 8:**
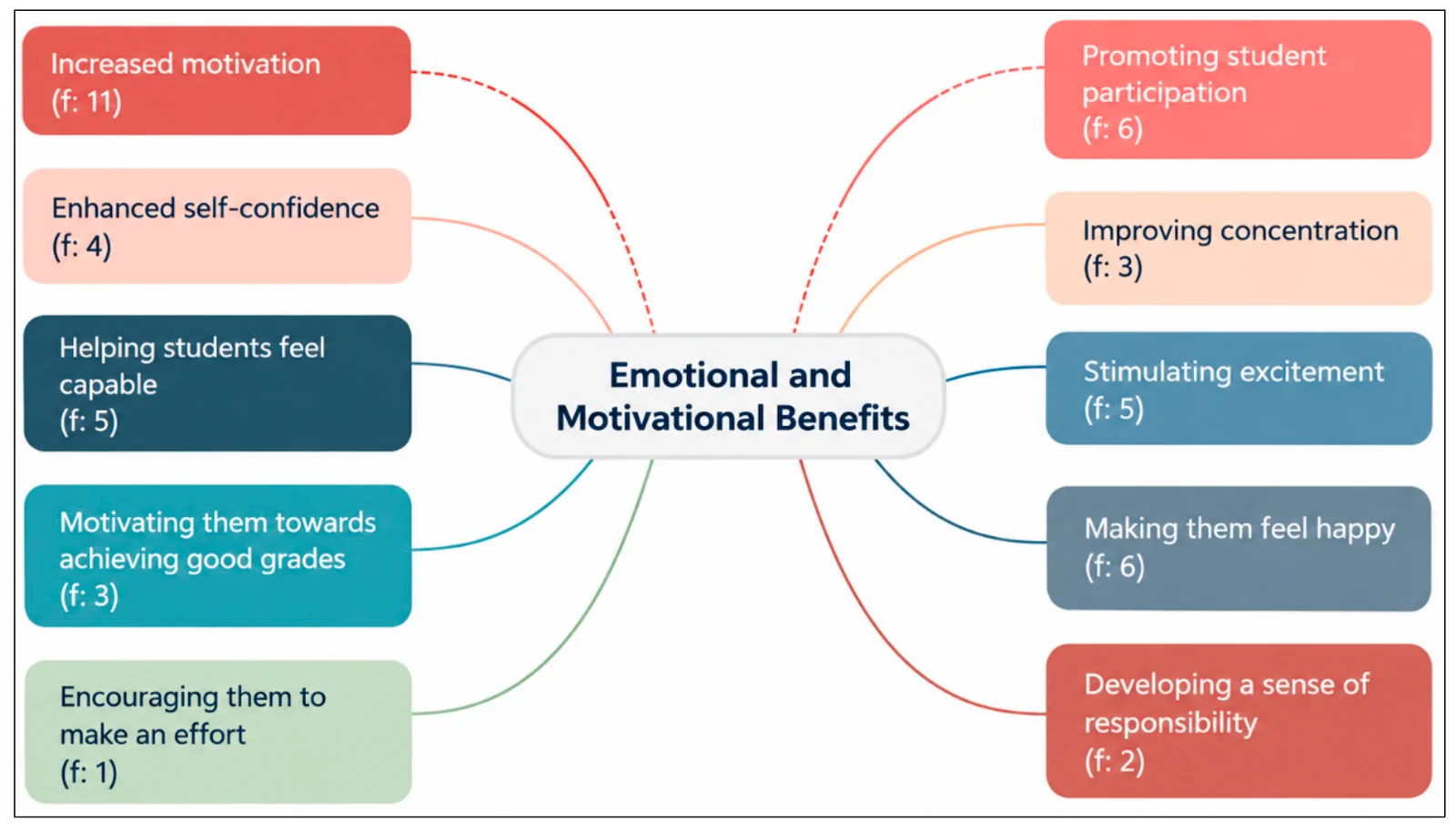
Emotional and Motivational Benefits. Note. The colors of the boxes and their corresponding connecting lines are used only to visually distinguish the identified themes. The colors do not indicate different categories, levels, or relationships among the themes. The abbreviation “f” indicates the frequency of the corresponding theme.

The “Historical and Cultural Awareness” sub-theme encompasses codes such as fostering cultural awareness, teaching cultural heritage through visual means, enabling the establishment of connections within a historical and cultural context, developing an awareness of spatial history, helping students understand the relationship between the past and the present, interpreting social events within a spatial context, enabling the spatial interpretation of population distribution, and conducting socio-cultural structure analysis (Figure 8). Among these, enabling the establishment of connections within a historical and cultural context (f = 5) and conducting socio-cultural structure analysis (f = 5) were the most frequently identified codes. Fostering cultural awareness (f = 4), teaching cultural heritage through visual means (f = 4), and developing an awareness of spatial history (f = 4) were also frequently identified. In contrast, helping students understand the relationship between the past and the present (f = 3), interpreting social events within a spatial context (f = 3), and enabling the spatial interpretation of population distribution (f = 3) were the least frequently identified codes. These findings suggest that participants perceived GIS as helping to make abstract historical knowledge more concrete. Examples of reference statements under this theme include: *“Population movements in large cities can be tracked more easily with Geographic Information Systems.”* M8 (p. 73) *“We learned that during the Persian War about the Battle of Thermopylae*, *a big part of that war was because it was a real narrow passage and if that wasn’t there*, *the war might have been a lot different. When we talked about it in class and read it in our textbooks*, *I never really thought about the geography of that place but then I looked it up playing around on Google Earth and really you can see how narrow the passage was back then. I mean on one side you have a huge mountain and on the other side is a road but I read [not on Google Earth] that the road is where the water line used to be back then.”* (T3, p. 119). These findings demonstrate that GIS is not merely a geographical tool but an effective instructional material for the spatial contextualization of historical and cultural data.

Within the “Emotional and Motivational Benefits” theme, the identified codes include increased motivation, enhanced self-confidence, helping students feel capable, motivating them towards achieving good grades, encouraging them to make an effort, promoting student participation, improving concentration, stimulating excitement, making them feel happy, and developing a sense of responsibility. Among these, increased motivation (f = 11) was the most frequently identified code, followed by promoting student participation (f = 6) and making them feel happy (f = 6). Helping students feel capable (f = 5) and stimulating excitement (f = 5) were also frequently identified. In contrast, encouraging them to make an effort (f = 1) was the least frequently identified code, followed by developing a sense of responsibility (f = 2). Examining participants in the intervention groups’ views that support this theme, *“Following the instructions. The steps you taught us were fun and easy for me. Everything was enjoyable*, *and being able to see the distribution of products by creating a symbology map was particularly enjoyable.”* (T7, s. 81) and *“Students often expressed their interest when the teacher showed recognizable places on the GIS maps by making comments like ‘Cool!’, ‘Wow!’ and ‘Goaaa!’”* (M4, p. 244) *“…I think the GIS program should be used in similar courses and schools. Because we students sometimes get bored in class. I think this program should be used in classes because it attracts our attention and emphasizes visuals. I think someone who doesn’t like geography can learn to like it this way. Normally*, *a student might find it difficult to open and read a book*, *but in a computer environment*, *it can become more interesting and fun. Because we spend most of our time in front of computers…”* (T8, p. 147), *“Using GIS was interesting … I found myself wanting to complete all the activities … it was exciting.”* (M6, p. 17), *“Learning about geography using a GPS was more fun than taking notes*.” (T6, p. 71), *“Because I like to use the computer I like to use GIS”* (M2, p. 145), *“It was a lot of fun. I had never used Google Earth before. I’m thinking of using it in my classes from now on because I think it will be very useful.”* (T2, p. 67)— expressions such as these demonstrate that participants in the intervention groups’ attitudes towards the learning process have shifted positively. The effect of GIS’s technical capabilities on learning motivation has been described in some studies as: *“I think nothing was hard because everything was fun and easy”* (T3, s. 214) and *“It is both more enjoyable*, *and here we draw the map ourselves. It also provides a lot of information. I don’t think it has any downsides.”* (T1, p. 122), “We were always studying before; thanks to these activities, our classes are more fun. I do not get bored but rather enjoy learning” (M3, p. 256)—a statement that points to the cross-disciplinary interest aroused by technology. In light of these findings, it can be concluded that GIS use in social studies lessons strengthens participant’ motivation towards academic achievement and contributes to the development of a multidimensional, positive attitude towards the subject.

**Figure 9:**
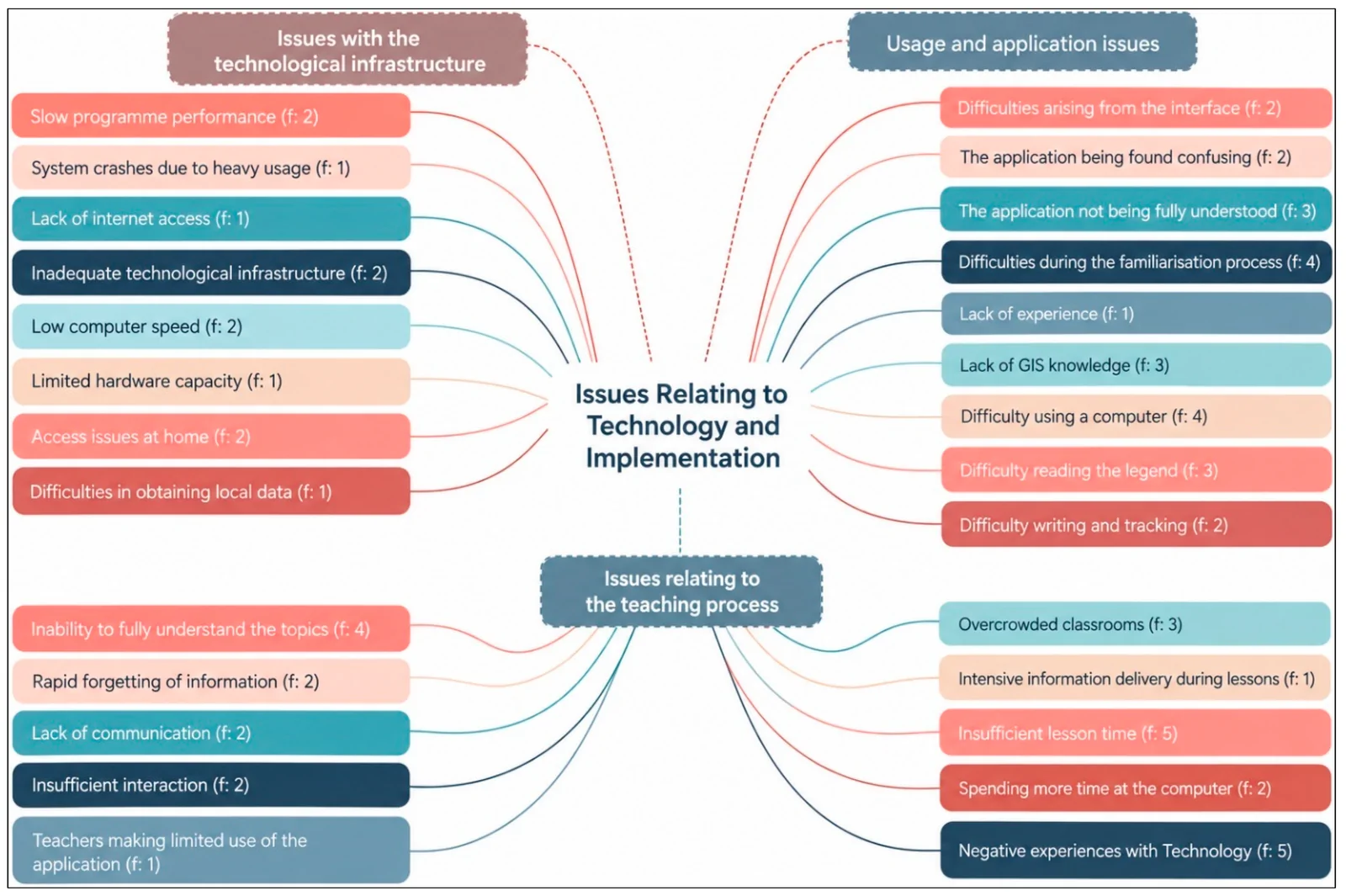
Issues Relating to Technology and Implementation. Note. The colors of the boxes and their corresponding connecting lines are used only to visually distinguish the identified themes. The colors do not indicate different categories, levels, or relationships among the themes. The abbreviation “f” indicates the frequency of the corresponding theme.

In accordance with the data presented in Figure 9, the constraints encountered in GIS use were categorized under three core sub-themes. The first sub-theme, “Issues with the Technological Infrastructure,” includes slow programme performance, system crashes due to heavy usage, lack of internet access, inadequate technological infrastructure, low computer speed, limited hardware capacity, access issues at home, and difficulties in obtaining local data. Among these, slow programme performance (f = 2), inadequate technological infrastructure (f = 2), low computer speed (f = 2), and access issues at home (f = 2) were the most frequently identified codes. In contrast, system crashes due to heavy usage (f = 1), lack of internet access (f = 1), limited hardware capacity (f = 1), and difficulties in obtaining local data (f = 1) were the least frequently identified codes. When the reference expressions are examined, the statement; “*If I were to use GIS in the future*, *I would arrange the computer laboratory to suit me. I would set up the cables and organize the desks so students could see me more comfortably. I would position myself closer to the projector so my voice could carry*, *or I would wear a lapel microphone so everyone could hear me when teaching GIS. Because not everyone can benefit from it—particularly those sitting at the back of the computer room*.” (T5, p. 84).

The second sub-theme, “Usage and Application Issues,” encompasses codes such as difficulties arising from the interface, the application being found confusing, the application not being fully understood, difficulties during the familiarisation process, lack of experience, lack of GIS knowledge, difficulty using a computer, difficulty reading the legend, and difficulty writing and tracking. Among these, difficulties during the familiarisation process (f = 4) and difficulty using a computer (f = 4) were the most frequently identified codes. These were followed by the application not being fully understood (f = 3), lack of GIS knowledge (f = 3), and difficulty reading the legend (f = 3). In contrast, lack of experience (f = 1) was the least frequently identified code. Examining the reference statements pertaining to participants in the intervention groups’ experiences, *“It was sort of hard*, *sort of confusing because like you have to click like different buttons and then you have added on and all that.”* (M1, p. 9) and *“I found the ‘Learning GIS’ activity difficult*, *because it was my first time using GIS.”* (M5, p. 50). “This program is suitable for our level, I just had a little difficulty drawing the map.” (T1, p. 120). These statements indicate that the technical nature of GIS creates cognitive load in the initial stages.

The third sub-theme, “Issues Relating to the Teaching Process,” encompasses codes such as inability to fully understand the topics, rapid forgetting of information, lack of communication, insufficient interaction, teachers making limited use of the application, overcrowded classrooms, intensive information delivery during lessons, insufficient lesson time, spending more time at the computer, and negative experiences with technology. Among these, insufficient lesson time (f = 5) and negative experiences with technology (f = 5) were the most frequently identified codes, followed by inability to fully understand the topics (f = 4) and overcrowded classrooms (f = 3). In contrast, teachers making limited use of the application (f = 1) and intensive information delivery during lessons (f = 1) were the least frequently identified codes. Reference statements from the studies covered under this theme include: “*In social studies*, *I normally could not follow along because the classroom was very noisy; I had some difficulty because of the high noise level*”, *“I cannot keep track of where we are in the textbook*, *and with GIS we cover topics faster than in the book.”* (M5 p. 50), *“I just couldn’t find places I wanted; it was hard to navigate*, *I felt confused sometimes”* (T6, p. 157)—statements drawing attention to large class sizes and other negative aspects of the process. Similarly, in response to the question “*What problems did students encounter most often while working on GIS applications?*” the following responses were recorded: *“Computers running slowly”*, *“Insufficient time”*, *“Teachers sometimes not wanting to allow it”*, *“The computer laboratory being too small”*, *“Forgetting things very quickly” and “Not being able to use it at home.”* (T4, p. 167)—a statement that emphasizes the inadequacy of technical preparation time and lesson duration.

## 4. Conclusions and Discussion

In this study, which aimed to examine the effect of GIS use in social studies on students’ academic achievement, the data obtained through document analysis were analyzed within a mixed-meta methodological framework using meta-analytic and meta-thematic analysis techniques.

### 4.1. Quantitative Findings

A total of 17 national and international studies examining GIS use in social studies and meeting the established inclusion criteria were included in the meta-analysis within the scope of this research. The findings indicate that, on the basis of the effect size classification proposed by Thalheimer and Cook (2002), the pooled random-effects estimate indicated a large positive effect; however, the high heterogeneity and wide prediction interval warrant cautious interpretation of the magnitude and generalizability of this effect. This result suggests that GIS can be considered an effective instructional tool that supports academic achievement in social studies teaching. The findings are also consistent with various national (Aladağ, 2010; İneç, 2012; Şimşek, 2007; Ünal & Sarı, 2012; Ünlü & Yıldırım, 2016, among others) and international (Adeleke, 2017; Alazmi, 2025; Mayben, 2010, among others) studies in the literature. Furthermore, a study by Değirmenci and Altaş (2016) established that GIS-assisted instruction in higher education geography courses positively influenced students’ academic achievement. Similarly, Öner and Aydın (2014) concluded that GIS use in ninth-grade Geography classes increased students’ academic achievement. It is also evident that the effects of GIS use are not confined to academic achievement alone; studies conducted across different disciplines have demonstrated that GIS applications also exert positive effects on student attitudes (Artvinli, 2010; Bikar et al., 2022; Demirci, 2011; Meydan & Öner, 2014, among others). These findings demonstrate that GIS makes meaningful contributions not only to cognitive outcomes but also to the affective domain.

Among the 17 studies examining academic achievement, the overall effect of GIS use was estimated, followed by moderator analyses examining whether effect sizes differed according to four moderator variables: sample size, grade level, intervention duration, and publication type.

Regarding the sample size moderator, although the effect sizes differed across the small-, medium-, and large-sample groups, the difference between the groups was not statistically significant. Descriptively, the highest effect size was observed in the small-sample group; however, this difference does not indicate a statistically significant moderating effect. This finding suggests that sample size did not significantly differentiate the effect of GIS use on academic achievement in the current dataset. Nevertheless, some studies have reported that instructional interventions conducted with medium-sized samples may be more effective in terms of student achievement (Değirmenci, 2015; Koca et al., 2017). The findings of the present meta-analysis, however, indicate that the descriptive differences observed across sample-size groups were not statistically significant. Therefore, sample size does not appear to be a substantial moderator explaining variation in the effect sizes within the current dataset.

Regarding the grade-level moderator, the analysis indicated that the difference between the groups was not statistically significant. Descriptively, the highest effect size was observed at the sixth-grade level, whereas the lowest effect size was observed among students in grades 7–8. However, these differences should not be interpreted as indicating that grade level significantly alters the effect of GIS use on academic achievement. The fact that studies conducted by Daşdemir (2013) and Mayben (2010) have reported different findings across grade levels suggests that grade level may be a study characteristic worth considering when evaluating the effectiveness of GIS applications. Nevertheless, the findings of the present meta-analysis do not support the conclusion that the observed differences across grade levels are statistically significant. Therefore, when interpreting the findings related to grade level, the number of studies within each subgroup and the corresponding confidence intervals should also be taken into consideration.

Regarding the intervention duration moderator, the analysis indicated that the differences among the effect sizes observed across the different intervention-duration groups were not statistically significant. Although the highest effect size was descriptively observed for interventions lasting 1–4 weeks, there was no evidence that intervention duration significantly differentiated the effect sizes. Nevertheless, the descriptive differences observed across intervention durations should not be entirely disregarded. Indeed, studies conducted by Adeleke (2017) and Aladağ (2010) have shown that short-term GIS interventions can have positive effects on academic achievement. The findings of the present meta-analysis likewise indicate that a higher effect size was observed for short-term interventions; however, this difference was not statistically significant. Therefore, although intervention duration was not identified as a significant moderator in the present meta-analysis, it may be useful to consider the effect sizes obtained across different intervention durations in conjunction with the characteristics of the interventions.

Regarding the publication type moderator, although effect sizes differed across the journal article, master’s thesis, and doctoral dissertation groups, the difference between the groups was not statistically significant. Descriptively, higher effect sizes were observed in thesis studies compared with journal articles. However, these differences cannot be interpreted as indicating that publication type significantly alters the effect of GIS use on academic achievement. Therefore, there is insufficient statistical evidence in the current dataset to conclude that publication type is a significant moderator. Nevertheless, it should be acknowledged that studies published in different formats may differ in terms of sample characteristics, implementation conditions, research designs, and reporting practices. Accordingly, the effect sizes obtained across publication types should be interpreted in conjunction with other study characteristics.

Overall, the meta-analysis findings indicate that the use of GIS in social studies education is an effective instructional tool that supports participants in the intervention groups’ academic achievement. However, the moderator analyses revealed no statistically significant differences in effect sizes with respect to sample size (Q = 0.382, *p* = .826), grade level (Q = 3.082, *p* = .214), intervention duration (Q = 1.309, *p* = .520), or publication type (Q = 2.977, *p* = .226). Although some descriptive differences were observed across the subgroups, these differences should not be interpreted as statistically significant moderating effects. In particular, the limited number of studies in some subgroups warrants caution when interpreting the moderator findings.

The I^2^ value of 88.172% obtained from the meta-analysis indicates a high level of heterogeneity among the studies. The fact that none of the four moderator variables examined was statistically significant suggests that the observed high heterogeneity cannot be adequately explained by these variables. However, this finding does not imply that these variables have no relationship with heterogeneity. Characteristics not examined as moderators in the present study, such as sample characteristics, the content and format of GIS applications, implementation conditions and duration, the measurement instruments used, teacher characteristics, and other methodological differences, may have contributed to the variation in effect sizes across studies. Thus, although the present findings support a positive effect of GIS use on academic achievement, they also indicate that the sources of the high heterogeneity observed across studies cannot be explained solely by the moderator variables examined. Future research should examine different sample characteristics, implementation conditions, forms of GIS use, and research methodologies in greater detail to contribute to a more comprehensive understanding of the conditions under which the effect of GIS use on academic achievement may vary.

### 4.2. Qualitative Findings

As the study was designed as a mixed-meta-methods study, the qualitative component was incorporated into the process to complement the findings of the quantitative synthesis and to provide a broader perspective on the use of GIS in social studies education. Accordingly, the qualitative component was examined through meta-thematic analysis, and the findings were organized under six themes: “Higher-Order Thinking and Cognitive Skill Development,” “The Learning Process and Contributions to the Learning Environment,” “Map Literacy and the Development of Spatial Skills,” “Awareness,” “Emotional and Motivational Benefits,” and “Issues Relating to Technology and Implementation.”

The first prominent theme identified in the meta-thematic analysis was higher-order thinking and cognitive skill development. The studies included in the qualitative synthesis reported that GIS use contributed positively to, or supported, various cognitive processes and skills. In this context, digital literacy, research, critical thinking, communication, inquiry, problem identification and solving, comparison, inference, synthesis, comprehension, and interpretation emerged as prominent skills. In addition, some studies indicated that GIS-supported learning enabled participants in the intervention groups to evaluate historical and geographical events from different perspectives, relate the knowledge they acquired to everyday life, and establish connections across different topics and disciplines. These findings are consistent with the views of Audet and Ludwig (2000), who suggested that GIS use can involve higher-order thinking processes and emphasized the importance of inquiry- and problem-based approaches in developing such processes. Similarly, Liu et al. (2010) reported differences in students’ levels of higher-order thinking in GIS-supported learning environments, while Shin (2007) highlighted the potential of GIS to activate different forms of thinking and map skills. Taken together, the qualitative findings suggest that GIS-supported learning environments can provide opportunities for students to engage in higher-order cognitive processes. However, these findings should not be interpreted as evidence of objective and generalizable improvements in cognitive skills within the current synthesis, but rather as contributions and experiences reported in qualitative studies.

The second theme was the learning process and contributions to the learning environment. Qualitative studies associated GIS use with meaningful learning experiences, individualized learning opportunities, discussion-based learning, collaborative learning, and learning processes extending beyond the classroom. GIS was also reported to facilitate access to information, make instructional materials more concrete and comprehensible, support observation, and facilitate students’ engagement with learning tasks. For example, Baloğlu Uğurlu (2013) reported that GIS-supported instruction provided students with meaningful learning experiences and was perceived as more effective than traditional lecture-based instruction. Shin (2003) emphasized students’ opportunities to independently process and manage data, whereas Storie (2000) associated GIS applications with active participation and collaborative learning. Özgen and Oban Çakıcıoğlu (2008) suggested that GIS-supported applications may be compatible with constructivist learning environments. Overall, these findings indicate that the qualitative literature generally conceptualizes GIS as a technology that enriches the learning environment and diversifies students’ learning experiences. However, it is not possible to conclude that these learning experiences were responsible for the academic achievement effect identified in the present meta-analysis. Rather, these findings provide contextual complementary evidence for the quantitative finding on academic achievement.

The third theme was map literacy and the development of spatial skills. Qualitative studies indicated that GIS use was positively associated with participants in the intervention groups’ perceptions, geographical orientation, understanding of their local and global environments, and abilities to read, interpret, and use maps. Participants were reported to have opportunities to visualize and analyze spatial information through GIS, and these processes enabled them to approach geographical phenomena in a more relational and holistic manner. Previous research has also highlighted the potential of GIS-supported learning for spatial thinking and map skills (Lee & Bednarz, 2009; Hickman, 2023). In a study employing both quantitative and qualitative data, Alazmi (2025) similarly reported greater gains in spatial thinking skills among students receiving GIS-supported instruction compared with those receiving traditional instruction. The findings of the present qualitative synthesis are generally consistent with this literature and indicate that GIS use can provide positive experiences and perceived contributions related to spatial and map skills. However, because spatial thinking and map skills were not among the outcomes synthesized in the quantitative meta-analysis of the present study, these findings should not be interpreted as evidence of a general or causal effect of GIS on spatial skills.

Another theme identified in the study was awareness. The qualitative literature indicated that GIS-supported activities were positively associated with participants in the intervention groups’ geographical, historical, and cultural awareness. In particular, the use of maps, spatial data, and visualization tools provided students with opportunities to examine geographical and historical events from different perspectives and to evaluate places, events, and cultural characteristics within their spatial contexts. Shin (2003) and Songer (2007) highlighted the role of GIS in students’ understanding of geographical and historical phenomena. Similarly, Egiebor and Foster (2018) suggested that story maps can help students explore historical and cultural places and visualize these places within their geographical contexts. Thus, the qualitative findings suggest that GIS-supported activities can provide learning opportunities for examining geographical, historical, and cultural phenomena within their broader contexts. Nevertheless, these findings should not be regarded as evidence of a quantitatively measured and generalizable increase in levels of awareness.

The fifth theme was emotional and motivational benefits. Qualitative studies reported various positive findings regarding participants in the intervention groups’ interest, motivation, self-confidence, attention, classroom participation, and emotional responses to GIS use. Participants described GIS activities as interesting, enjoyable, motivating, and interactive; some studies also reported positive experiences concerning classroom participation, academic effort, and perceptions of competence. These findings are consistent with previous research highlighting the potential of GIS-supported learning to enhance students’ intrinsic motivation and interest in learning (Aladağ, 2010; Kerskı, 2000; Sasaki et al., 2008). Ünal and Sarı (2012) likewise reported that students enjoyed GIS-based activities and that their classroom participation increased. However, the affective and motivational findings should be distinguished from the quantitative academic achievement findings of the present study. Motivation, participation, and positive learning experiences may represent important contextual factors in the learning process; however, the present synthesis did not test whether these variables mediated the effect on academic achievement. Therefore, these findings were interpreted not as mechanisms underlying the quantitative academic achievement effect, but as complementary qualitative evidence concerning participants in the intervention groups’ experiences of GIS-supported learning.

The final theme was issues relating to technology and implementation. The studies included in the meta-thematic analysis reported various challenges associated with the classroom use of GIS, including inadequate technological infrastructure, limited access to computers and hardware, insufficient instructional time, technical problems, and pedagogical constraints. Similar challenges have also been identified in previous research. Ramhadani et al. (2025) noted structural and technological difficulties in GIS applications, while Şimşek (2007) emphasized that limited instructional time and inadequate computer laboratory facilities can make the implementation of GIS applications more difficult. These findings suggest that the feasibility of GIS-supported instruction and its potential contributions to the learning process may be influenced by implementation conditions. However, because these implementation characteristics were not systematically and sufficiently reported in the quantitative studies, whether they function as moderators of the effect on academic achievement could not be directly tested within the present meta-analysis.

Overall, the meta-thematic findings indicate that the qualitative literature associates GIS-supported learning with engagement in cognitive processes, enriched learning experiences, spatial and map skills, geographical and historical awareness, and positive affective and motivational experiences. At the same time, technological and implementation-related challenges may constrain the realization of these opportunities. These qualitative findings complement the quantitative results demonstrating a positive overall effect of GIS use on academic achievement by providing contextual information about how GIS is experienced across different learning environments and what types of contributions have been reported. However, a causal relationship extending from cognitive or affective processes to academic achievement should not be inferred from the two forms of evidence. In particular, no direct study-level correspondence was established between the qualitative themes and effect sizes, and the role of these themes as moderators or mediators of academic achievement was not tested. Therefore, the qualitative findings should be interpreted not as causal mechanisms underlying the quantitative academic achievement effect, but as contextual and potentially explanatory perspectives on GIS-supported learning.

### 4.3. Integration of Quantitative and Qualitative Results

When the quantitative and qualitative findings are considered together, the two analytical components appear to be complementary, although the functions of the two types of evidence differ. The meta-analysis provides quantitative evidence indicating that GIS use has a large positive effect on participants in the intervention groups’ academic achievement in social studies courses. In contrast, the meta-thematic analysis reveals how GIS-supported learning has been experienced and described in the qualitative research literature. Within this context, various contributions have been reported in relation to higher-order thinking and cognitive processes, meaningful and active learning experiences, map and spatial skills, geographical, historical, and cultural awareness, as well as students’ motivation, engagement, and self-confidence.

Therefore, rather than directly explaining the causal basis of the quantitatively identified effect on academic achievement, the qualitative findings provide contextual information that broadens the interpretation of this finding. In particular, findings concerning cognitive, spatial, learning-process-related, and affective contributions demonstrate the range of learning experiences and reported outcomes associated with GIS use in qualitative studies. These findings may contribute to a contextual interpretation of the positive effect on academic achievement identified in the meta-analysis. However, they should not be interpreted as indicating that these factors caused or mediated the effect on academic achievement. In the present study, no direct study-level correspondence was established between the qualitative themes and quantitative effect sizes, and no mediation analysis was conducted to determine whether cognitive, spatial, or affective processes explain differences in academic achievement.

The technological and implementation-related challenges identified in the qualitative synthesis also contribute to a contextual understanding of GIS-supported learning. These findings indicate that GIS applications may encounter various practical and contextual constraints, including technological infrastructure, instructional time, and implementation conditions. However, because these characteristics were not adequately and systematically reported in the quantitative studies, their role in explaining differences in academic achievement or modifying effect sizes could not be directly examined within the present synthesis.

Accordingly, the integration of the two types of evidence should be understood not as a causal explanatory pathway, but as the combination of two complementary perspectives. While the quantitative synthesis suggests an overall positive effect of GIS use on academic achievement, the qualitative synthesis highlights the range of learning experiences, perceived contributions, and implementation conditions reported in GIS-supported social studies instruction. Taken together, these findings provide a more comprehensive understanding of the phenomenon under investigation while maintaining the distinction between quantitatively demonstrated effects and qualitatively reported experiences and outcomes.

## 5. Limitations and Recommendations for Future Research

This study has several limitations. First, the relatively limited number of studies included in the meta-analysis and the small number of studies in some moderator categories may have reduced the statistical power of the moderator analyses and limited the generalizability of the findings. Specifically, the grade-level moderator included only two studies contributing three effect sizes in the fifth-grade group, the intervention-duration moderator included three studies in the group with interventions lasting eight weeks or longer, and the publication-type moderator included three studies in the doctoral dissertation group. Therefore, the absence of statistically significant moderator differences should not be interpreted as definitive evidence that these variables have no role in the effectiveness of GIS use. Differences among the primary studies in research design, sample characteristics, measurement instruments, and intervention procedures also warrant caution when interpreting the pooled effect sizes.

A second limitation concerns the substantial heterogeneity observed across the studies (I^2^ = 88.172%). This heterogeneity may be associated with differences in sample characteristics, grade level, intervention duration, GIS-based instructional practices, instructional approaches, and control-group practices. However, none of the moderators examined statistically accounted for a significant proportion of the observed variation. Thus, other study- and implementation-related characteristics may contribute to the heterogeneity and should be examined in future research.

Another limitation is the insufficient and inconsistent reporting of teachers’ GIS knowledge, experience, technological competence, and implementation characteristics. Consequently, potentially important factors such as teacher preparation, professional development, teacher guidance, instructional roles, and implementation fidelity could not be examined as moderators. Similarly, limited reporting of hardware resources, Internet access, and GIS software prevented a systematic examination of the role of technological infrastructure. Future primary studies should therefore provide more detailed information on teacher preparation, implementation processes, technological conditions, and GIS tools.

The study protocol was also not registered in PROSPERO or another publicly accessible registration platform before the literature search and analysis. Nevertheless, the inclusion and exclusion criteria, search procedures, and analytical approach are described in detail in the Methods section. Future meta-analyses should consider preregistering their protocols to enhance transparency and reproducibility.

Furthermore, restricting the search to selected databases, academic platforms, and publication types may have resulted in some relevant studies being overlooked. Although theses and dissertations were included, publication bias cannot be completely excluded. The funnel plot, Egger’s regression test (*p* = .078), and Fail-Safe N (N = 1056) were considered collectively. While these results did not provide statistically significant evidence of substantial publication bias, they should not be interpreted as demonstrating its complete absence.

Finally, the meta-analysis focused primarily on academic achievement in social studies education and therefore did not comprehensively examine other potential outcomes of GIS use, such as motivation, attitudes, critical thinking, problem-solving, and spatial thinking. Future research should investigate a broader range of learning outcomes and systematically report teacher characteristics, implementation fidelity, technological infrastructure, and pedagogical approaches to clarify the conditions under which GIS applications are most effective.

## Figures and Tables

**Table 1:** Inclusion and Exclusion Criteria.

Criterion	Quantitative Component: Meta-Analysis	Qualitative Component: Meta-Thematic Analysis
**Research Topic**	Studies examining the use of Geographic Information Systems (GIS) in social studies education and their impact on participants in the intervention groups’ academic achievement	Studies examining the use of Geographic Information Systems (GIS) in social studies education and qualitative findings related to the teaching–learning process
**Educational Context**	Studies conducted within the scope of social studies education	Studies conducted within the scope of social studies education
**Research Design**	Studies that use an experimental or quasi-experimental design and include an experimental/control or comparison group	Mixed-methods studies containing qualitative or quantitative data
**Participants**	Participants enrolled in social studies courses and assigned to intervention groups.	Participants in the intervention groups receiving GIS-supported social studies instruction
**Results/Data** **Requirements**	Studies that provide sufficient statistical data (e.g., mean, standard deviation, and sample size, or statistics that can be converted into these) to enable the calculation of effect sizes related to academic achievement	Studies presenting qualitative findings regarding participants’ views, experiences, perceptions, contributions, or challenges related to the use of GIS
**Publication Type**	Peer-reviewed journal articles and master’s and doctoral theses	Peer-reviewed journal articles and master’s and doctoral theses
**Language**	In Turkish or English	In Turkish or English
**Full-Text Access**	Studies for which the full text is available	Studies for which the full text is available
**Exclusion Criteria**	Studies that do not examine the use of GIS in social studies education; do not include experimental or quasi-experimental comparisons; do not assess academic achievement; do not provide sufficient statistical data to calculate effect sizes; and duplicate studies	Studies that do not examine the use of GIS in social studies education; that do not contain qualitative findings related to the research questions; from which qualitative findings cannot be extracted for analysis; and duplicate studies

**Table 2:** Dataset Used in the Meta-Analysis Process.

References	Method	Sample Size	Grade Level	Weeks	Publication Type
(Ünal & Sarı, 2012)	Quantitative	49	5th grade	6 weeks	Article
(Kaya & Aydın, 2011)	Quantitative	40	6th grade	3 weeks	Article
(Güneş et al., 2020)	Mixed	60	5th grade	6 weeks	Article
(Şimşek, 2007)	Mixed	68	6th grade	6 weeks	PhD Thesis
(İneç, 2012)	Quantitative	143	6th grade	3 weeks	Master’s Thesis
(Keskin, 2018)	Mixed	100	University	14 weeks	PhD Thesis
(Baloğlu Uğurlu, 2013)	Quantitative	40	6th grade	5 weeks	PhD Thesis
(Uzun, 2025)	Quantitative	46	6th grade	10 weeks	Master’s Thesis
(Sönmez, 2019)	Quantitative	41	6th grade	6 weeks	Master’s Thesis
(Adeleke, 2017)	Quantitative	126	5th grade	4 weeks	Article
(Aladağ, 2010)	Quantitative	44	7th grade	3 weeks	Article
(Koca et al., 2017)	Quantitative	66	6th grade	6 weeks	Article
(Daşdemir, 2013)	Quantitative	139	6th grade	3 weeks	Master’s Thesis
(Türel, 2019)	Quantitative	52	7th grade	5 weeks	Master’s Thesis
(Meechandee & Meekaew, 2025)	Quantitative	30	8th grade	8 weeks	Article
(Yayla, 2019)	Mixed	47	6th grade	6 weeks	Article
(Alazmi, 2025)	Quantitative	232	8th grade	5 weeks	Article

**Table 3:** Dataset Used in the Meta-Thematic Analysis Process.

Code	References	Grade Level	Data Collection Method	Contributions to Themes
T1	Aladağ (2010)	7th grade	Observation form and Interview	A1, A5, A6
M1	Egiebor and Foster (2018)	8th grade	Interview	A4, A6
M2	Baloğlu Uğurlu (2013)	middle school	Survey	A1, A5
M3	Güneş et al. (2020)	5th grade	Interview	A4, A5
M4	Shin (2007)	4th grade	Interview, Field notes and Video recordings	A5
T2	Yayla (2019)	6th grade	Interview	A1, A4, A5
T3	Tesar (2010)	7th grade	Interview	A4
M5	Sönmez and Akbaş (2019)	6th grade	Interview	A1, A2, A6
T4	Şimşek (2007)	6th grade	Interview	A2, A6
T5	Keskin (2018)	university	Interview	A6
M6	Alazmi (2025)	8th grade	Interview	A4, A5
T6	Mayben (2010)	7th grade	Interview	A4, A5, A6
T7	Aktürk (2012)	6th grade	Interview	A3, A5
M7	Ataç (2022)	university	Interview	A4
M8	Şimşek (2013)	university	Observation, Interview and Document analysis	A1, A4
M9	Ayas and Taştan (2016)	6th grade	Interview	A3
T8	Değirmenci (2015)	university	Interview and Student Journals	A3, A5

**Table 4:** Summary Results of the Meta-Analysis.

Model	N	ES	SE	95% CI Lower	95% CI Upper	Z	*p*	Q	df	*p*	I^2^	τ^2^
FEM	17	0.878	0.060	0.761	0.995	14.718	<0.001	135.268	16	<0.001	88.172	0.467
REM	17	1.021	0.179	0.671	1.372	5.705	<0.001					

Note. N = number of studies; ES = effect size; g = Hedges’ g; SE = standard error; CI = confidence interval; FEM = fixed-effect model; REM = random-effects model; Q = Cochran’s Q statistic; I^2^ = percentage of variability attributable to between-study heterogeneity.

**Table 5:** Moderator Analysis Results.

Groups		N	ES (g)	95% CI Lower	95% CI Upper	Z	*p*	Q	df	*p*
**Sample Size**	Small Sample	8	1.132	0.904	1.359	9.768	0.000	0.382	2	0.826
	Medium Sample	4	0.931	0.108	1.755	2.217	0.027			
	Large Sample	5	0.943	0.144	1.741	2.314	0.021			
**Grade Level**	Grade 5	3	0.775	−0.085	1.635	1.766	0.077	3.082	2	0.214
	Grade 6	9	1.244	0.841	1.646	6.055	0.000			
	Grade 7–8-graduate	4	0.610	−0.029	1.249	1.870	0.061			
**Duration (Weeks)**	1–4 Weeks	5	1.301	0.728	1.874	4.453	0.000	1.309	2	0.520
	5–7 Weeks	9	0.875	0.372	1.377	3.413	0.001			
	8+ Weeks	3	0.980	0.678	1.281	6.366	0.000			
**Publication Type**	Article	9	0.777	0.330	1.224	3.408	0.001	2.977	2	0.226
	Master’s Thesis	5	1.286	0.811	1.761	5.306	0.000			
	Doctoral Thesis	3	1.305	0.676	1.935	4.067	0.000			

**Note:** Since there was only one study at the university level, the class level was not included in the moderator analysis, and the analysis was conducted based on 16 studies.

## Data Availability

The data are available on request from the corresponding author.

## Supplementary Materials

The following supporting information can be downloaded at: https://www.mdpi.com/article/10.3390/jintelligence14100256/s1, Author code compatibility tables.

## Appendix A

**Table A1:** Inter-Coder Agreement Values for Themes and Codes in the Meta-Thematic Analysis.

**Higher-Order Thinking and** **Cognitive Skill Development**					**The Learning Process and Contributions to the Learning Environment**					**Map Literacy and Spatial Skill** **Development**				
	**A2**					**A2**					**A2**			
**A1**		+	−	Σ	**A1**		+	−	Σ	**A1**		+	−	Σ
	+	14	1	15		+	11	0	11		+	18	1	19
	−	1	12	13		−	2	11	13		−	2	17	19
	Σ	15	13	28		Σ	13	11	24		Σ	20	18	38
**Kappa: 0.85 *p*: .000**					**Kappa: 0.83 *p*: .000**					**Kappa: 0.84 *p*: .000**				
**Awareness**					**Affective and Motivational** **Contributions**					**Technological and** **Implementation Challenges**				
	**A2**					**A2**					**A2**			
**A1**		+	−	Σ	**A1**		+	−	Σ	**A1**		+	−	Σ
	+	18	1	19		+	12	1	11		+	28	2	30
	−	1	15	16		−	1	10	11		−	3	25	28
	Σ	19	16	35		Σ	13	10	24		Σ	31	27	58
**Kappa: 0.88 *p*: .000**					**Kappa: 0.83 *p*: .000**					**Kappa: 0.82 *p*: .000**				
