# Supplementary material for: The Effectiveness of Geographic Information Systems in Social Studies Education: A Mixed-Meta Synthesis of Academic Achievement and Qualitative Learning Outcomes
Source: J Intell. 2026 Oct 12;14(10):256. doi: 10.3390/jintelligence14100256 (PMC13705788; doi:10.3390/jintelligence14100256)
Supplement: Supplementary file 1 [file jintelligence-14-00256-s001.zip › jintelligence-4476843-supplementary.pdf]

## AUTHOR CODE COMPATIBILITY TABLES

***Table 1***

| 1) HIGHER-ORDER THINKING AND COGNITIVE SKILL DEVELOPMENT | A1 | A2 | Adaptation |
|---|---|---|---|
| Developing critical thinking skills | + | + | ✓ |
| Developing inquisitive thinking skills | + | + | ✓ |
| Developing problem-solving skills | + | + | ✓ |
| Equipping students with problem-identification skills | + | − | ✗ |
| Developing reasoning skills | + | + | ✓ |
| Developing interpretation skills | + | + | ✓ |
| Promoting the ability to compare and contrast | − | − | ✓ |
| Developing synthesis skills | + | + | ✓ |
| Enhancing the ability to understand and interpret information | − | − | ✓ |
| Enabling evaluation of events within historical and geographical context | + | + | ✓ |
| Facilitating the transfer of knowledge to real-life contexts | − | − | ✓ |
| Enabling connections between subjects | + | + | ✓ |
| Promoting research competence | − | − | ✓ |
| Developing digital literacy skills | + | + | ✓ |
| Fostering effective communication competence | − | − | ✓ |

**NOT:** The codes initially listed in Table 1 were systematically re-evaluated through a joint reconciliation process to address discrepancies between the independent reviewers. Mutually agreed modifications were subsequently applied to align non-conforming codes, and the final revised versions have been updated in Table 2.

***Table 2***

| Original Version | Final version |
|---|---|
| Promoting the ability to compare and contrast | Developing comparison skills |
| Enhancing the ability to understand and interpret information | Developing comprehension skills |
| Facilitating the transfer of knowledge to real-life contexts | Enabling application of knowledge to everyday life |
| Promoting research competence | Developing research skills |
| Fostering effective communication competence | Developing communication skills |

*Table 3*

| 2) CONTRIBUTIONS TO THE LEARNING PROCESS AND LEARNING ENVIRONMENT | A1 | A2 | Adaptation |
|---|---|---|---|
| Promoting meaningful learning | + | + | ✓ |
| Supporting individual learning | + | + | ✓ |
| Supporting learning through discussion | + | + | ✓ |
| Promoting collaborative learning experiences | − | − | ✓ |
| Supporting out-of-class learning | + | + | ✓ |
| Fostering continuous learning | − | − | ✓ |
| Creating a dynamic learning environment | + | + | ✓ |
| Enabling access to information | + | − | ✗ |
| Making teaching materials concrete and understandable | + | + | ✓ |
| Promoting learning through observation | − | − | ✓ |
| Supporting effective feedback processes | − | − | ✓ |

**NOT:** The codes initially listed in Table 3 were systematically re-evaluated through a joint reconciliation process to address discrepancies between the independent reviewers. Mutually agreed modifications were subsequently applied to align non-conforming codes, and the final revised versions have been updated in Table 4.

*Table 4*

| Original Version | Final version |
|---|---|
| Promoting collaborative learning experiences | Encouraging group learning |
| Fostering continuous learning | Supporting lifelong learning |
| Promoting learning through observation | Supporting observation |
| Supporting effective feedback processes | Facilitating the provision of feedback |

*Table 5*

| 3) MAP LITERACY AND SPATIAL SKILL DEVELOPMENT | A1 | A2 | Adaptation |
|---|---|---|---|
| Developing spatial awareness | + | + | ✓ |
| Developing the ability to locate places | + | + | ✓ |
| Developing the ability to find one's way | + | + | ✓ |
| Developing the ability to judge spatial scale | + | − | ✗ |
| Being able to analyse spatial distribution | + | + | ✓ |
| Identifying spatial patterns | + | + | ✓ |
| Observing spatial change | − | − | ✓ |
| Fostering a holistic perspective on space | + | + | ✓ |
| Promoting a holistic perspective on physical and human dimensions | − | − | ✓ |
| Teaching the map–scale relationship | + | + | ✓ |
| Teaching different types of maps | + | + | ✓ |
| Developing the ability to use map display elements | + | + | ✓ |
| Facilitating map customization, annotation, and spatial manipulation | − | − | ✓ |
| Enabling interactive mapping | + | + | ✓ |
| Fostering story map development and spatial narrative creation | − | − | ✓ |
| Supporting the creation of maps with different content | + | + | ✓ |
| Developing map presentation skills | + | + | ✓ |
| Developing graphic design skills | − | − | ✓ |
| Enhancing cartographic design and visual | + | + | ✓ |

**NOT:** The codes initially listed in Table 5 were systematically re-evaluated through a joint reconciliation process to address discrepancies between the independent reviewers. Mutually agreed modifications were subsequently applied to align non-conforming codes, and the final revised versions have been updated in Table 6.

*Table 6*

| Original Version | Final version |
|---|---|
| Analyzing spatial-temporal dynamics | Observing spatial change |
| Promoting a holistic perspective on physical and human dimensions | Ensuring a holistic approach to natural and human dimensions of space |
| Facilitating map customization, annotation, and spatial manipulation | Enabling modifications and annotations on maps |
| Fostering story map development and spatial narrative creation | Enabling the creation of story maps |
| Enhancing cartographic design and visual communication skills | Developing graphic design skills |

*Table 7*

| 4) THE AWARENESS THEME<br>4.1) Geographical Awareness | A1 | A2 | Adaptation |
|---|---|---|---|
| Fostering environmental awareness | + | + | ✓ |
| Fostering awareness of natural resources | + | + | ✓ |
| Developing a global perspective | + | + | ✓ |
| Promoting cross-national and global spatial awareness | − | − | ✓ |
| Introducing landforms and land cover | + | + | ✓ |
| Fostering conceptual understanding of climatic patterns and dynamics | − | − | ✓ |
| Teaching the locations of lakes and rivers | + | + | ✓ |
| Introducing geographical patterns | + | + | ✓ |
| Enhancing student engagement with immediate spatial and local surroundings | − | − | ✓ |
| Providing opportunities to study different population groups | + | + | ✓ |
| **4.2) Historical and Cultural Awareness** | | | |
| Fostering cultural awareness | + | + | ✓ |
| Leveraging visual cartography and media to convey cultural heritage | − | − | ✓ |
| Establishing connections within a historical and cultural context | + | + | ✓ |
| Developing awareness of spatial history | + | + | ✓ |
| Helping students understand the relationship between the past and the present | + | − | ✗ |
| Interpreting social events within a spatial context | + | + | ✓ |
| Enabling spatial interpretation of population distribution | + | − | ✗ |
| Facilitating multi-scalar socio-cultural structure analysis | − | − | ✓ |

**NOT:** The codes initially listed in Table 7 were systematically re-evaluated through a joint reconciliation process to address discrepancies between the independent reviewers. Mutually agreed modifications were subsequently applied to align non-conforming codes, and the final revised versions have been updated in Table 8.

*Table 8*

| Original Version | Final version |
|---|---|
| Promoting cross-national and global spatial awareness | Introducing different countries |
| Fostering conceptual understanding of climatic patterns and dynamics | Helping students understand climate characteristics |
| Enhancing student engagement with immediate | Enabling students to establish connections |

| Original Version | Final version |
|---|---|
| spatial and local surroundings | with their local environment |
| Leveraging visual cartography and media to convey cultural heritage | Teaching cultural heritage through visual means |
| Facilitating multi-scalar socio-cultural structure analysis | Conducting socio-cultural structure analysis |

***Table 9***

| 5) AFFECTIVE AND MOTIVATIONAL CONTRIBUTIONS | A1 | A2 | Adaptation |
|---|---|---|---|
| Increased motivation | + | + | ✓ |
| Enhanced self-confidence | + | + | ✓ |
| Helping students feel capable | + | + | ✓ |
| Enhancing academic achievement motivation and performance orientation | – | – | ✓ |
| Encouraging them to make an effort | + | + | ✓ |
| Promoting student participation | + | – | ✗ |
| Sustaining cognitive focus and attentional capacity | – | – | ✓ |
| Stimulating excitement | + | + | ✓ |
| Promoting positive affective states and academic well-being | – | – | ✓ |
| Developing a sense of responsibility | + | + | ✓ |

**NOT:** The codes initially listed in Table 9 were systematically re-evaluated through a joint reconciliation process to address discrepancies between the independent reviewers. Mutually agreed modifications were subsequently applied to align non-conforming codes, and the final revised versions have been updated in Table 10.

***Table 10***

| Original Version | Final version |
|---|---|
| Enhancing academic achievement motivation and performance orientation | Motivating them towards achieving good grades |
| Sustaining cognitive focus and attentional capacity | Improving concentration |
| Promoting positive affective states and academic well-being spatial and local surroundings | Making them feel happy with their local environment |

*Table 11*

| 6) TECHNOLOGICAL AND IMPLEMENTATION CHALLENGES<br>6.1) Issues Related to the Technological Infrastructure | A1 | A2 | Adaptation |
|---|---|---|---|
| Slow programme performance | + | + | ✓ |
| System crashes due to heavy usage | + | + | ✓ |
| Lack of internet access | + | + | ✓ |
| Inadequate technological infrastructure | + | − | ✗ |
| Hardware performance limitations and processing constraints | − | − | ✓ |
| Limited hardware capacity | + | + | ✓ |
| Domestic digital divide and out-of-school accessibility barriers | − | − | ✓ |
| Difficulties in obtaining local data | + | + | ✓ |
| **6.2) Topics Related to the Teaching Process** | | | |
| Inability to fully understand the topics | + | + | ✓ |
| Rapid forgetting of information | + | − | ✗ |
| Lack of communication | + | + | ✓ |
| Insufficient interaction | − | − | ✓ |
| Teachers making limited use of the application | + | + | ✓ |
| Overcrowded classrooms | + | + | ✓ |
| Intensive information delivery during lessons | + | + | ✓ |
| Suboptimal levels of pedagogical and interpersonal interaction | − | − | ✓ |
| Spending more time at the computer | − | + | ✗ |
| Negative experiences with technology | + | + | ✓ |

**NOT:** The codes initially listed in Table 11 were systematically re-evaluated through a joint reconciliation process to address discrepancies between the independent reviewers. Mutually agreed modifications were subsequently applied to align non-conforming codes, and the final revised versions have been updated in Table 12.

*Table 12*

| Original Version | Final version |
|---|---|
| Hardware performance limitations and processing constraints | Low computer speed |
| Domestic digital divide and out-of-school accessibility barriers | Access issues at home |
| Suboptimal levels of pedagogical and interpersonal interaction | Insufficient interaction |
